# funRiceGenes dataset for comprehensive understanding and application of rice functional genes

**DOI:** 10.1093/gigascience/gix119

**Published:** 2017-12-04

**Authors:** Wen Yao, Guangwei Li, Yiming Yu, Yidan Ouyang

**Affiliations:** 1 National Key Laboratory of Crop Genetic Improvement, National Center of Plant Gene Research, Huazhong Agricultural University, Wuhan 430070, China; 2 College of Life Sciences, Henan Agricultural University, Zhengzhou 450002, China

**Keywords:** *Oryza sativa* (rice), functional genomics, interaction network, genetic improvement

## Abstract

**Background:**

As a main staple food, rice is also a model plant for functional genomic studies of monocots. Decoding of every DNA element of the rice genome is essential for genetic improvement to address increasing food demands. The past 15 years have witnessed extraordinary advances in rice functional genomics. Systematic characterization and proper deposition of every rice gene are vital for both functional studies and crop genetic improvement.

**Findings:**

We built a comprehensive and accurate dataset of ∼2800 functionally characterized rice genes and ∼5000 members of different gene families by integrating data from available databases and reviewing every publication on rice functional genomic studies. The dataset accounts for 19.2% of the 39 045 annotated protein-coding rice genes, which provides the most exhaustive archive for investigating the functions of rice genes. We also constructed 214 gene interaction networks based on 1841 connections between 1310 genes. The largest network with 762 genes indicated that pleiotropic genes linked different biological pathways. Increasing degree of conservation of the flowering pathway was observed among more closely related plants, implying substantial value of rice genes for future dissection of flowering regulation in other crops. All data are deposited in the funRiceGenes database (https://funricegenes.github.io/). Functionality for advanced search and continuous updating of the database are provided by a Shiny application (http://funricegenes.ncpgr.cn/).

**Conclusions:**

The funRiceGenes dataset would enable further exploring of the crosslink between gene functions and natural variations in rice, which can also facilitate breeding design to improve target agronomic traits of rice.

## Background

Rice is a main staple food that feeds half of the world's population. Improvement of yield and resistance to multiple biotic and abiotic stresses of rice are essential strategies to cope with the increasing world population and the diminishing arable land. Decoding the genetic reservoirs of rice is the basis for rice phenotype improvement.

Functional genomic studies in model organisms have made great contributions to the studies of a wide range of other species [1]. In the last decade, the functions of a number of rice genes were explored with the availability of the genome sequence of *Oryza sativa* L. ssp. *japonica* cv. Nipponbare [2]. Genes controlling important agronomic traits, including grain yield [3, 4], blast [5] and blight [6, 7] disease resistance, insect resistance [8], and abiotic stress resistance [9, 10], were functionally characterized. Some of these genes were utilized in rice breeding directly based on marker-assisted strategy and CRISPR (Clustered Regularly Interspaced Short Palindromic Repeats) [11–13]. Moreover, the putative homologs of some rice genes were investigated in other crops such as wheat [14–17], barley [18], and maize [19]. As rice is an ideal model of the grass family, characterization of rice genes would greatly facilitate genomic studies and molecular breeding in other crops.

Abundant information on functionally characterized genes of *Arabidopsis* is archived in The Arabidopsis Information Resource (TAIR) [20], while a list of functionally characterized maize genes are integrated in the maizeGDB database [21], which greatly promoted the functional genomics studies in plants. Detailed information on *Drosophila* genes stored in the FlyBase database is of great value to the studies in *Drosophila* and humans [22]. The rice genome annotation project maintained by the Michigan State University [23] and the Rice Annotation Project Database (RAP-DB) [24] greatly promoted the progress of rice functional genomics. Although a number of curated rice genes are collected in RAP-DB and Oryzabase [25], not all the functionally characterized rice genes are properly deposited in existing databases. In the long term, the functions of all rice genes will be decoded [26]. As a result, a comprehensive archive of all functionally characterized rice genes involved in diverse pathways with live updating is urgently in demand.

In this study, we constructed a comprehensive, up-to-date database of rice functional genes, which includes ∼2800 cloned rice genes and ∼5000 members of different gene families. Interaction networks comprising 1310 functionally characterized rice genes were constructed, which revealed complex regulation and crosstalk of different biological pathways. We also developed a Shiny application that allows easy addition of newly reported rice genes. As far as we are concerned, this is the most comprehensive and accurate database of functionally characterized rice genes with continuous updating.

## Results

### Collection of functionally characterized rice genes

A database [27] maintained by the China Rice Data Center collects information on thousands of cloned rice genes in Chinese. Information on these genes was downloaded using in-house R scripts, including gene symbol, publications, the corresponding gene model in the Nipponbare reference genome, and a brief summary of the corresponding gene. The abstract, the author affiliation, and the full text of each publication were subsequently extracted from the PubMed database. Next, we manually curated the dataset based on the full text of each publication and obtained 1297 functionally characterized rice genes.

We further downloaded 29 982 publication records by querying the PubMed database with the keyword rice ((rice[Title] OR rice[Title/Abstract]), data until 13 February 2014). All records were grouped by published journal. After removing the records involved in the China Rice Data Center and ones irrelevant to rice functional genomics, the full texts of the remaining publications were downloaded and reviewed, which identified 441 additional functionally characterized rice genes. Information on each gene, including the GenBank accession number and the corresponding gene model in the Nipponbare genome, was extracted.

As an integrated rice science database, Oryzabase [25] also provides information on a portion of functionally characterized rice genes with manual curation. We downloaded 10 140 records comprising a list of genes from this database [28], and 5531 records with assigned Nipponbare genomic locus were retained. After removing of redundant records in datasets obtained from the other 2 approaches, 469 functionally characterized genes excluding members of gene families were retrieved. All information on the 469 genes was manually curated based on the review of research publications. Finally, 2207 functionally characterized rice genes were collected until 13 February 2014.

We further collected ∼3600 members of various gene families by integrating data from the Rice Genome Annotation Project database [29], the Oryzabase database, and research publications. All the data were deposited in the funRiceGenes database [30].

A Shiny application [31] was then developed to facilitate utilization of this dataset, which also enabled easy addition of newly reported genes to the database. New genes were added to this database using the Shiny application, based on daily email alerts of search results from the PubMed database with the keyword rice (rice[Title] OR rice[Title/Abstract]) [32]. For all PubMed records in the email alert, we identified ones on functionally characterized rice genes. We then went over the full publication of each record and identified the gene symbol and gene model in the reference genome. After inputting the gene symbol, the gene model in the reference genome, and the PubMed identifier, the Shiny application will fetch the corresponding publication record from PubMed and extract key information automatically. We also kept track of new records in the database of Oryzabase and China Rice Data Center, which were then added to our database using the Shiny application. Since 13 February 2014, funRiceGenes has been updated every 2 weeks using the Shiny application. All updated records are available at the NEWS menu of the funRiceGenes database [33]. As of 23 February 2017, ∼2800 functionally characterized genes and ∼5000 gene family members were archived in the funRiceGenes database, which accounted for 19.2% of the 39 045 annotated protein-coding rice genes (Additional file 1: Table S1; Additional file 2: Table S2) [33, 23].

### Overview of the dataset regarding functionally characterized rice genes

Rice functional genomic studies developed rapidly after the public availability of the Nipponbare reference genome (Additional file 3: Fig. S1). In total, ∼3553 publications with respect to ∼2800 functionally characterized genes were collected (Additional file 4: Table S3). These publications came from more than 215 journals, 31.0% of which were published in *The Plant Journal, Plant Physiology, Plant Molecular Biology, The Plant Cell, Molecular Plant*, and *New Phytologist* (Additional file 4: Table S3). Among all published papers, 4 words—rice, gene, protein, and expression—were observed with the highest frequency in titles, while the words rice, gene, expression, protein, plant, mutant, and stress were found with the highest frequency in the abstract (Additional file 5: Fig. S2; Additional file 6: Fig. S3). More than 1800 affiliations from all over the world contributed to rice functional genomic studies (Additional file 7: Table S4), and scientists from China, Japan, Korea, United States, and India accounted for the majority of the progress (Additional file 8: Fig. S4).

**Figure 1:**
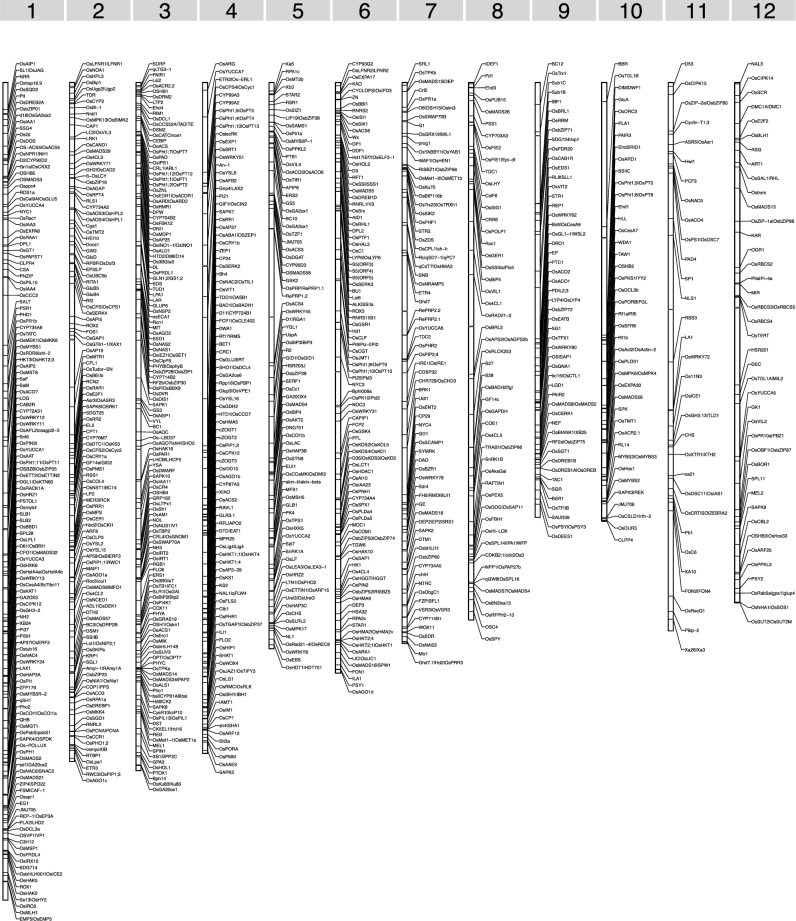
Chromosome distribution of representative functionally characterized rice genes. The chromosomes are represented as vertical rectangles, and each horizontal line denotes the position of a functionally characterized rice gene. Symbols of all genes are labeled. A total of 930 representative genes are shown.

Genomic positions were determined for more than 98.1% of all functionally characterized rice genes based on the corresponding gene models of the Nipponbare reference genome (Additional file 1: Table S1; Fig. 1). Twenty-five genes were absent from or showed substantial sequence divergence relative to the Nipponbare reference genome, and their genomic positions were determined based on the reference genome sequences of *indica* varieties Zhenshan 97 and Minghui 63 [34]. The remaining 24 genes could not be located in the genome, which was likely due to the sequence divergence between different rice germplasms.

A number of genes were investigated simultaneously by distinct research groups based on various rice accessions, mutants, or phenotypic traits. As a result, 637 genes were assigned more than 1 symbol (Additional file 1: Table S1). In contrast, the same symbols were sometimes assigned to different genes due to the lack of communication (Additional file 9: Table S5).

Based on the concurrence of gene symbols and keywords regarding phenotype description or biological process in the same sentence of an abstract or a title in the literature, the functions of corresponding genes were summarized with manual curation. A total of 441 keywords were investigated, which generated 21 872 records for 1952 genes (Additional file 10: Table S6). Among all 441 keywords, yield and grain yield were found in 311 records for 115 genes, while grain width, grain length, grain weight, and grain size were detected in 139 records for 53 genes. Among all 77 genes retrieved with a heading date or flowering time, 13 were also associated with yield or grain yield. Likewise, 7 genes involved in iron utilization, phosphate uptake, and sugar transporting were related to grain yield. We also found that 335 genes were involved in different stress signaling pathways, while 139 genes were related to rice diseases, including blast, bacterial blight, and sheath blight.

**Figure 2:**
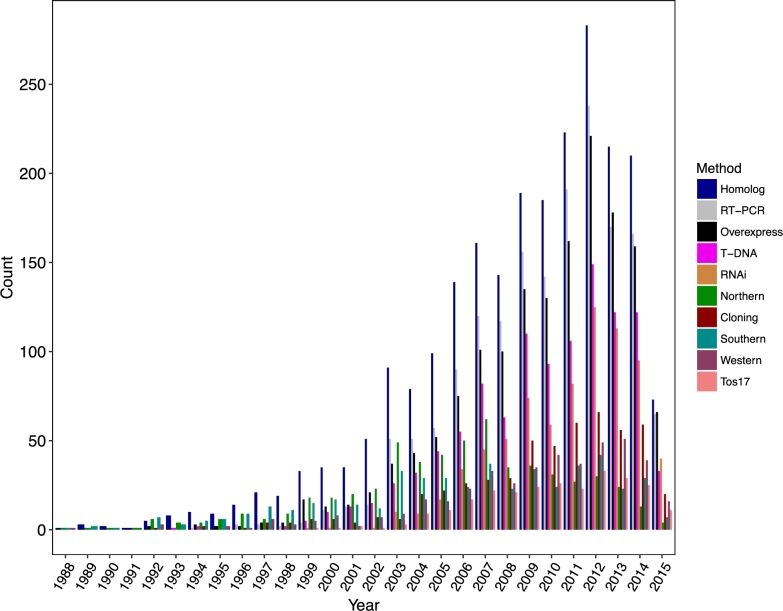
Usage of various biotechniques in rice functional genomics studies. The y-axis indicates the number of publications using a specific biotechnique. Data after 18 June 2015 are not shown.

Progress in rice functional genomics benefited from the development of various technologies and the availability of diverse genomic and genetic resources. We found that homolog information was the most frequently used resource in rice functional genomics studies, and reverse transcriptase polymerase chain reaction was the most commonly used technique to analyze gene expression level (Fig. 2). Overexpression or RNAi were frequently used to disturb gene expression, which contributed to the dissection of the association between gene expression and phenotype variation. Creation of mutants using T-DNA and Tos17 insertions contributed significantly to rice gene cloning, while GWAS (genome-wide association study) and CRISPR became new strategies to dissect the functions of rice genes in recent years [35, 36].

### Interaction networks of functionally characterized rice genes

Physical and genetic interactions between different rice genes were frequently reported. However, a global view of the interaction networks for all functionally characterized rice genes remains to be elaborated. We constructed interaction networks of functionally characterized genes based on the concurrence of the symbols of 2 or more genes in the same sentence of an abstract or a title of research publications using in-house R script with manual curation. A sentence in which 2 or more genes were observed was regarded as evidence supporting the connection between these genes. In total, 1841 connections supported by 4046 evidences were detected, which comprised 1310 genes constituting 214 interaction networks (Additional file 11: Table S7).

**Figure 3:**
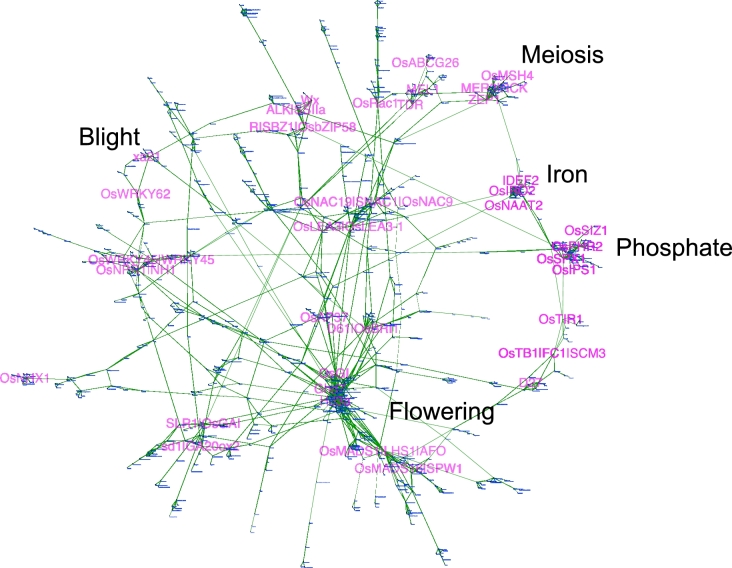
The gene interaction network comprising 762 genes. Each white node represents a functionally characterized rice gene, and gene symbols are marked beside the node. Each green edge indicates a connection between 2 genes. Genes involved in the same biological pathways are indicated.

The largest network was composed of 762 genes including ones associated with flowering, phosphate uptake and homeostasis, iron uptake, stress signaling, blight disease resistance, meiosis, BR (brassinosteroid) and GA (gibberellin) signaling, grain weight, and endosperm development (Fig. 3). Genes related to the same trait were clustered together, indicating the trustworthiness of this approach. The enormous size of this network was mainly caused by pleiotropic genes involved in different biological pathways. For example, *Ghd8* was responsible for grain number, plant height, and heading date [37]. *Ghd8* connected to genes controlling heading date including *Ehd1* [38], *Hd16* [39], and *RFT1* [38], and genes controlling tillering including *MOC1* [40], which was further connected with *MIP1*, a gene regulating tillering and plant height [41]. The other 213 interaction networks were made up of 548 rice genes, 88% of which contained only 2 or 3 genes (Additional file 12: Fig. S5). The second largest network contained 14 genes involved in glutamine metabolism, including *OsAMT1;3, GAD3*, and *GAT1* [42, 43]. Genes in terms of small RNA biogenesis including *OsDCL3a, OsDCL1*, and *OsHEN1* were observed in a 10-gene network (Additional file 12: Fig. S5) [44–46].

**Figure 4:**
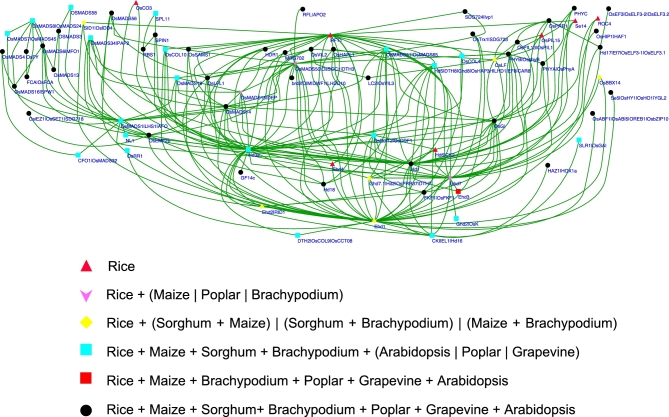
Interaction network of genes regulating flowering in rice and the orthologs of these genes in other plants. Each node represents a functionally characterized rice gene. Each edge indicates a connection between 2 genes. Genes with different number of orthologs are indicated with different colors and shapes. “Rice + (Maize | Poplar)” indicates “Rice and Maize” or “Rice and Poplar.” Detailed information is shown in Additional file 13: Table S8.

We further constructed an interaction network using 77 genes involved in flowering regulation (Fig. 4). Based on the orthologous groups among 7 plants provided by the Rice Genome Annotation Project [47], we found that 40 of the 77 genes had orthologous genes in sorghum, maize, Brachypodium, Arabidopsis, poplar, and grapevine, and orthologous genes were also identified for another 20 rice genes in sorghum, maize, and Brachypodium (Fig. 4; Additional file 13: Table S8). Only 7 genes—*RFT1, Ehd4, Hd6, OsCO3, ROC4, Se14*, and *OsPIL15*—were unique to rice. These results demonstrated the increasing degree of conservation of the flowering pathway among plants with closer phylogenetic relationships, implying substantial value of knowledge on functionally characterized rice genes to future dissection of flowering time regulation in other crops.

## Discussion

In this study, we built a comprehensive and accurate database of functionally characterized rice genes, funRiceGenes, which provides a valuable resource for rice functional genomic studies. funRiceGenes was constructed by integrating data from PubMed, Oryzabase, and China Rice Data Center, and it has been updated every 2 weeks using a Shiny application. For each gene in the funRiceGenes database, the gene symbol, the genomic locus in the reference genome, and the published papers on this gene were identified. Compared with Textpresso for *Oryza sativa* [48], which is a comprehensive collection of literatures on rice, we further built the associations between genomic locus or symbol of genes and literatures [49]. Based on the literature identified for each gene, we summarized the brief functions of each gene and constructed interaction networks for all genes. The evidence supporting the functions of all collected genes and the interaction networks are unique to the funRiceGenes database. In addition, a user-friendly query interface and tidy data for downloading are provided in the funRiceGenes database.

Along with the sequence and phenotype data of thousands of rice accessions reported in recent years, the affluent information of rice genes in our database would enable further exploring of the crosslink between gene functions and natural variations. We found that a cloned rice gene *OsSGL* (LOC_Os02g04130, chr02:1799733-1800811), which regulated grain weight in rice, was ∼70 kb away from a GWAS peak (chr02:1871732) in terms of grain weight [50, 51]. Likewise, another gene *OsPPKL3* (LOC_Os12g42310, chr12:26273157-26282197), which regulated grain length, is ∼90 kb away from a GWAS peak (chr12:26182880) associated with grain length [52, 53]. The functions of *OsSGL* and *OsPPKL3* were characterized by transgenic studies, and the natural variations of the 2 genes are yet to be dissected.

Our database is also beneficial to the interpretation of large-scale DNA, mRNA, and other sequencing datasets in rice. Analyses of these data usually identify differentially expressed genes, gene co-expression networks, differentially methylated regions, and ChIP-seq peaks, etc. The detailed information concerning the several thousands of rice genes archived in this database would be helpful for illustration of these results [54]. Batch query functions are provided, allowing search of this database with multiple genes belonging to a pathway/biological process or defined gene set. Our work in rice would facilitate functional genomic studies of other crops including wheat, sorghum, and maize.

Pyramiding and editing of functionally characterized rice genes regulating important agronomic traits by molecular marker–assisted selection and CRISPR are 2 promising approaches used to breed new rice varieties in recent years [55–57]. Thus, this database would play an important role in future rice breeding. For a specific agronomic trait, all related genes could be retrieved from this database conveniently for further manipulation [58]. For any of these genes, all relevant publications and a brief summary are available in this database [59]. The sequences of different alleles reported are also archived in this database. These resources would greatly facilitate breeding design to improve target agronomic traits by pyramiding of elite alleles or knocking out deleterious alleles. In addition, the effect of 1 gene might be enhanced or masked by other genes [60]. Thus, the gene interaction networks provided in this database could also be taken into account when making breeding designs.

## Materials and Methods

### Geocoding of author affiliations

The latitudes and longitudes of all the author affiliations were obtained using the application interface provided by the DATASCIENCETOOLKIT website [61] with in-house R scripts. For author affiliations that failed to be geocoded at high resolutions, we further used the Mapeasy website [62] to find the accurate latitudes and longitudes. The R package ggmap was used to demonstrate the positions of all affiliations on the world map [63].

### Extraction of information from PDF files

The occurrence of keywords, including map-based cloning, positional cloning, accession number, accession No., northern blot, northern analysis, northern hybridization, and the regular expression “os[0-1][0-9]g[0-9]+.*” in PDF files were inspected utilizing the R tm [64] package.

### Construction of interaction networks

The R package igraph [65] was used to build the interaction networks based on all the connection information between genes. The networks were then exported in a data format suitable for Cytoscape, which was used to visualize the network [66].

## Availability of supporting source code and requirements

Project name: funRiceGenes (funRiceGenes, RRID:SCR_015778)

Project home page: http://funricegenes.ncpgr.cn/

GitHub repository: https://github.com/venyao/RICENCODE

Operating system(s): platform independent

Programming language: R (≥3.1.0)

Other requirements: tested with R packages shiny (1.0.5), shinythemes (1.1.1), shinyBS (0.61), RCurl (1.95.4.8), XML (3.98.1.9), stringr (1.2.0), plyr (1.8.4)

License: GPLv3

Any restrictions to use by nonacademics: none

Research resource ID: funRiceGenes, RRID:SCR_015778

## Availability of supporting data

A snapshot of the version of the funRiceGenes source code used in this paper is archived in the *GigaScience* repository, *Giga*DB [67].

## Additional files

Additional file 1: Table S1: A comprehensive list of functionally characterized rice genes.

Additional file 2: Table S2: List of rice gene families.

Additional file 3: Figure S1: Number of papers on rice functional genomic studies published in each year.

Additional file 4: Table S3: Publications on functionally characterized rice genes.

Additional file 5: Figure S2: Word cloud analysis of the titles of all the publications on rice functional genomic studies.

Additional file 6: Figure S3: Word cloud analysis of the abstracts of all the publications on rice functional genomic studies.

Additional file 7: Table S4: The geocoding results of author affiliations.

Additional file 8: Figure S4: Global distribution of affiliations contributed to rice functional genomics studies. All the affiliations are marked on the world map as blue circles based on their longitudes and latitudes. The size of the circle represents the number of publications conducted by each affiliation. Data after 18 June 2015 are not shown.

Additional file 9: Table S5: Genes with different functions that were assigned the same symbols.

Additional file 10: Table S6: Concurrence of the gene symbols and the keywords regarding phenotype description or biological process in the same sentence of the abstracts or titles of articles.

Additional file 11: Table S7: Concurrence of the symbols of 2 or more genes in the same sentence of the abstracts or titles of research publications.

Additional file 12: Figure S5: Gene interaction networks constructed based on the concurrence of 2 or more genes in the same sentence of abstracts or titles of publications. Each white node represents a gene, while each green edge indicates a connection between 2 genes.

Additional file 13: Table S8: Orthologs of genes regulating heading date in rice.

## Abbreviations

BR: brassinosteroid; CRISPR: Clustered Regularly Interspaced Short Palindromic Repeats; GA: gibberellin; GWAS: genome-wide association study; kb: kilo base; RAP-DB: the Rice Annotation Project Database; TAIR: the Arabidopsis Information Resource.

## Conflicts of interest

The authors declare that they have no competing interests.

## Author contributions

W.Y. conceived and designed the experiments. W.Y., G.L., Y.Y., and Y.O. analyzed the data. W.Y. and Y.O. wrote the paper.

## Funding

This research was supported by grants from the National Key Research and Development Program of China (2016YFD0100903), the National Natural Science Foundation of China (31771873 and 31371599), and the National Program for Support of Top-notch Young Professionals.

## Supplementary Material

GIGA-D-17-00154_Original_Submission.pdf

GIGA-D-17-00154_Revision_1.pdf

GIGA-D-17-00154_Revision_2.pdf

Response_to_Reviewer_Comments_Original_Submission.pdf

Response_to_Reviewer_Comments_Revision_1.pdf

Reviewer_1_Report_(Original_Submission) -- Andy Pereira 13 Aug 2017 Reviewed

Reviewer_2_Report_(Original_Submission) -- Takeshi Itoh 16 Aug 2017 Reviewed

Reviewer_2_Report_(Revision_1) -- Takeshi Itoh 23 Oct 2017 Reviewed

Supplement materials
