# Supplementary material for: funRiceGenes dataset for comprehensive understanding and application of rice functional genes
Source: Gigascience. 2017 Dec 4;7(1):gix119. doi: 10.1093/gigascience/gix119 (PMC5765555; doi:10.1093/gigascience/gix119)
Supplement: GIGA-D-17-00154_Original_Submission.pdf [file gix119_giga-d-17-00154_original_submission.pdf]

GigaScience

funRiceGenes dataset for comprehensive understanding and application of rice functional genes

--Manuscript Draft--

**Manuscript Number:**	GIGA-D-17-00154
**Full Title:**	funRiceGenes dataset for comprehensive understanding and application of rice functional genes
**Article Type:**	Research
**Funding Information:**	National Natural Science Foundation of China (31371599) — Dr. Yidan Ouyang<br>National Natural Science Foundation of China (31201190) — Not applicable<br>Outstanding Young Talents Program — Dr. Yidan Ouyang
**Abstract:**	Background: As a main staple food, rice is also a model plant for functional genomics researches of monocots. Decoding of every DNA element of the rice genome is essential for genetic improvement to address the increasing food demands. The past 15 years have witnessed extraordinary advances in rice functional genomics. Systematic characterization and proper deposition of every rice gene are vital for both functional studies and crop genetic improvement.<br>Findings: We built a comprehensive and accurate dataset of ~2,800 functionally characterized rice genes and ~5,000 members of different gene families, by integrating data from available database and review of every publication of rice functional genomics researches. The dataset accounts for 19.2% of the 39,045 annotated protein-coding rice genes, which provides the most exhaustive archive for investigating the functions of rice genes. We also constructed 214 gene interaction networks based on 1,841 connections between 1,310 genes. The largest network with 762 genes indicated that pleiotropic genes linked different biological pathways. Increasing degree of conservation of the flowering pathway was identified among closer related plants, implying substantial value for rice genes in future dissection of flowering regulation in other crops. All data are deposited in the funRiceGenes database (https://funricegenes.github.io/), with interactive query and continuous updating using a Shiny application (http://funricegenes.ncpgr.cn/).<br>Conclusions: The funRiceGenes dataset would enable further exploring of the crosslink between gene functions and natural variations in rice, which can also facilitate breeding design to improve target agronomic traits in rice.
**Corresponding Author:**	Yidan Ouyang<br><br>CHINA
**Corresponding Author Secondary Information:**	
**Corresponding Author's Institution:**	
**Corresponding Author's Secondary Institution:**	
**First Author:**	Wen Yao
**First Author Secondary Information:**	
**Order of Authors:**	Wen Yao<br>Guangwei Li<br>Yiming Yu<br>Yidan Ouyang
**Order of Authors Secondary Information:**	

Powered by Editorial Manager® and ProduXion Manager® from Aries Systems Corporation

**Opposed Reviewers:**	
**Additional Information:**	
**Question**	**Response**
Are you submitting this manuscript to a special series or article collection?	No
**Experimental design and statistics**<br><br>Full details of the experimental design and statistical methods used should be given in the Methods section, as detailed in our Minimum Standards Reporting Checklist. Information essential to interpreting the data presented should be made available in the figure legends.<br><br>Have you included all the information requested in your manuscript?	Yes
**Resources**<br><br>A description of all resources used, including antibodies, cell lines, animals and software tools, with enough information to allow them to be uniquely identified, should be included in the Methods section. Authors are strongly encouraged to cite Research Resource Identifiers (RRIDs) for antibodies, model organisms and tools, where possible.<br><br>Have you included the information requested as detailed in our Minimum Standards Reporting Checklist?	Yes
**Availability of data and materials**<br><br>All datasets and code on which the conclusions of the paper rely must be either included in your submission or deposited in publicly available repositories (where available and ethically appropriate), referencing such data using a unique identifier in the references and in the "Availability of Data and Materials" section of your manuscript.<br><br>Have you have met the above requirement as detailed in our Minimum Standards Reporting Checklist?	Yes

Manuscript Click here to download Manuscript Main.doc

funRiceGenes dataset for comprehensive understanding and application of rice functional genes

Wen Yao*, Guangwei Li, Yiming Yu, Yidan Ouyang*

National Key Laboratory of Crop Genetic Improvement, National Center of Plant Gene Research, Huazhong Agricultural University, Wuhan 430070, China

*Corresponding author: Wen Yao, ywhzau@gmail.com; Yidan Ouyang, diana1983941@mail.hzau.edu.cn

Abstract

Background: As a main staple food, rice is also a model plant for functional genomics researches of monocots. Decoding of every DNA element of the rice genome is essential for genetic improvement to address the increasing food demands. The past 15 years have witnessed extraordinary advances in rice functional genomics. Systematic characterization and proper deposition of every rice gene are vital for both functional studies and crop genetic improvement.

Findings: We built a comprehensive and accurate dataset of ~2,800 functionally characterized rice genes and ~5,000 members of different gene families, by integrating data from available database and review of every publication of rice functional genomics researches. The dataset accounts for 19.2% of the 39,045 annotated protein-coding rice genes, which provides the most exhaustive archive for investigating the functions of rice genes. We also constructed 214 gene interaction networks based on 1,841 connections between 1,310 genes. The largest network with

762 genes indicated that pleiotropic genes linked different biological pathways. Increasing degree of conservation of the flowering pathway was identified among closer related plants, implying substantial value for rice genes in future dissection of flowering regulation in other crops. All data are deposited in the funRiceGenes database (https://funricegenes.github.io/), with interactive query and continuous updating using a Shiny application (http://funricegenes.ncpgr.cn/).

Conclusions: The funRiceGenes dataset would enable further exploring of the crosslink between gene functions and natural variations in rice, which can also facilitate breeding design to improve target agronomic traits in rice.

Keywords: *Oryza sativa* (rice), functional genomics, interaction network, genetic improvement

Background

Rice is a main staple food that feeds half of the world population. Improvement of the yield and the resistance to multiple biotic and abiotic stresses of rice is an essential strategy to cope with the increasing world population and the diminishing arable land. Decoding of the genetic reservoirs hidden in the rice genome is the basis for rice phenotype improvement.

Functional genomic studies in model organisms have made great contributions to a wide range of other species [1]. In the last decade, the functions of a number of rice genes were explored with the availability of the genome sequence of *Oryza sativa* L.

ssp. *japonica* cv. Nipponbare [2]. Genes controlling important agronomic traits, including grain yield [3, 4], blast [5] and blight [6, 7] disease resistance, insect resistance [8], and abiotic stress resistance [9, 10], were functionally characterized. Some of these genes were utilized in rice breeding directly based on marker-assisted strategy and CRISPR [11-13]. Moreover, the putative homologs of some rice genes were investigated in other crops such as wheat [14-17], barley [18] and maize [19]. As an ideal model plant of the grass family, characterization of rice genes in the whole genome would greatly facilitate genomic researches and molecular breeding in other crops.

Abundant information on functionally characterized genes of *Arabidopsis* is archived in The Arabidopsis Information Resource (TAIR) [20], while a list of functionally characterized maize genes are integrated in the maizeGDB database (http://maizegdb.org/web_newgene.php?window=alltime), which greatly advance the functional genomics studies in plants. Detailed information on *Drosophila* genes stored in the FlyBase database (http://flybase.org) is of great value to the studies in *Drosophila* and human diseases [21]. The rice genome annotation project maintained by the Michigan State University of the USA [22] (http://rice.plantbiology.msu.edu/) and the National Agriculture and Food Research Organization of Japan [23] (http://rapdb.dna.affrc.go.jp/) greatly promotes the progress of rice functional genomics. However, most of the functionally characterized rice genes are not properly deposited in either of the two databases. In the long term, the functions of all rice genes will be decoded ultimately [24]. As a result, a comprehensive archive of all

functionally characterized rice genes involved in diverse pathways with live updating is urgently in demand.

In this study, we constructed a comprehensive database regarding rice functional genes up to date, which targets ~2,800 cloned rice genes and ~5,000 members of different gene families. Related interaction networks were constructed with respect to 1,310 functionally characterized rice genes, which revealed the complex regulation and crosstalk of different biological pathways. We also developed a Shiny application allowing easily addition of newly reported rice genes. As far as we are concerned, this is the most comprehensive and accurate database of functionally characterized rice genes with continuous updating.

Results

Collection of functionally characterized rice genes

A database (http://www.ricedata.cn/gene) maintained by the China Rice Data Center provides a part of information on rice genes in Chinese. Information on functionally characterized genes was downloaded using in-house R scripts, including the gene symbol, the publications, the corresponding gene model in the Nipponbare reference genome, and a brief summary of the corresponding gene. The abstract, the author affiliation, and the PDF file of each publication were subsequently extracted from the PubMed database. Next, we manually checked and revised the dataset based on the main context of each publication, which obtained 1,297 functionally characterized rice genes.

We further obtained 29,982 publication records by querying the PubMed database with the keyword "rice" ((rice[Title] OR rice[Title/Abstract]), data until 13 Feb 2014). All the records were grouped by the published journal. After removing of the records involved in the China Rice Data Center and ones irrelevant to rice functional genomics, the full texts of the remaining publications were downloaded and reviewed one by one, which identified additional 441 functionally characterized rice genes. Information on each gene, including the GenBank accession number and the corresponding gene model in the Nipponbare genome was extracted.

As an integrated rice science database, the Oryzabase (http://www.shigen.nig.ac.jp/rice/oryzabase/download/gene) also provides a portion of information on functionally characterized rice genes. We downloaded 10,140 records comprising a list of genes from this database (http://www.shigen.nig.ac.jp/rice/oryzabase/gene/download;jsessionid=52FB01A7F53441CF54F823AA1ED71DE0?classtag=GENE_EN_LIST), and 5,531 records with assigned Nipponbare genomic locus were retained. After removing of redundant records in datasets obtained from the other two approaches, 469 functionally characterized genes excluding members of gene families were retrieved. All the information on the 469 genes was manually checked based on the review of research publications. Finally, 2,207 functionally characterized rice genes were collected until 13 Feb 2014.

We further collected ~3,600 members of different gene families by integrating data from the database of Rice Genome Annotation Project

(http://rice.plantbiology.msu.edu/annotation_community_families.shtml), the Oryzabase database and research publications. All the data were deposited in the funRiceGenes database (https://funricegenes.github.io/).

A Shiny application (http://funricegenes.ncpgr.cn/) was developed to facilitate utilization of this information, which also enabled easy addition of newly reported genes to the database. New records could be added to this database using the Shiny application, based on daily email alert of the searching results from the PubMed database with the keyword “rice” (rice[Title] OR rice[Title/Abstract]). After inputting the gene symbol and gene model in the reference genome, the application will fetch the corresponding publication from PubMed and extract key information automatically. Till 23 Feb 2017, ~2,800 functionally characterized genes and ~5,000 gene family members were archived in the funRiceGenes database, which accounted for 19.2% of the 39,045 annotated protein-coding rice genes (Supplementary Table S1, Supplementary Table S2) (https://funricegenes.github.io/news/) [22].

Overview of the dataset regarding functionally characterized rice genes

Rice functional genomics got rapid development after the public availability of the Nipponbare reference genome sequence (Supplementary Figure S1). In total, about 3,553 publications with respect to ~2,800 functionally characterized genes were collected (Supplementary Table S3). These publications came from more than 215 journals, 31.0% of which were published in *The Plant Journal*, *Plant Physiology*, *Plant Molecular Biology*, *The Plant Cell*, *Molecular Plant*, and *New Phytologist* (Supplementary Table S3). Among all published papers, four words, rice, gene,

protein, and expression, showed the highest frequencies in titles, while the words including rice, gene, expression, protein, plant, mutant, and stress were found with the highest frequencies in the abstract (Supplementary Figure S2, Supplementary Figure S3). More than 1,800 affiliations from all over the world contributed to rice functional genomics researches (Supplementary Table S4), and scientists from China, Japan, Korea, USA and India accounted for the majority of the progress (Supplementary Figure S4).

Genomic positions were determined for more than 98.1% of all functionally characterized rice genes based on the corresponding gene models of the Nipponbare reference genome (Supplementary Table S1, Figure 1). Twenty-five genes were absent or showing substantial divergence in the *japonica* variety Nipponbare, and their genomic positions were determined based on the reference genome sequences of *indica* varieties Zhenshan 97 and Minghui 63 [25]. The remaining 24 genes were unable to be mapped on the genome, which was likely due to the sequence divergence between different rice germplasm.

A number of genes were investigated simultaneously by distinct research groups based on various rice accessions, mutants or phenotypic traits. As a result, 637 genes were assigned more than one symbol (Supplementary Table S1). In contrast, the same symbols were assigned to different genes due to the lack of communication (Supplementary Table S5).

Based on the concurrence of gene symbols and keywords regarding phenotype description or biological process in the same sentence of an abstract or a title in

literatures, the functions of corresponding genes were summarized with manual curation. A total of 441 keywords were investigated, which generated 21,872 records for 1,952 genes (Supplementary Table S6). The keywords “yield” and “grain yield” were found in 311 records for 115 genes, and the keywords of “grain width”, “grain length”, “grain weight” and “grain size” were detected in 139 records for 53 genes. Among all 77 genes retrieved with the keywords of “heading date” or “flowering time”, 13 were also associated with the keywords of “yield” or “grain yield”. Likewise, seven genes involved in iron utilization, phosphate uptake and sugar transporting were related to grain yield. We also found that 335 genes were involved in different stress signaling pathways, while 139 genes were in relation to rice diseases, including blast, bacterial blight, and sheath blight.

Progress in rice functional genomics benefited from the development of various technologies and diverse genomic and genetic resources. We found that homolog information was the most frequently used resource in rice functional genomics studies, and RT-PCR was the most commonly used technique to analyze gene expression level (Figure 2). Overexpression or RNAi were frequently used to disturb gene expression, which contributed to the rapid investigation of the relationship between gene expression and phenotype. Creation of mutants using T-DNA and Tos17 insertions contributed significantly to rice gene cloning, while GWAS and CRISPR became new strategies to dissect the functions of rice genes in recent years [26, 27].

Interaction networks of functionally characterized rice genes

Physical and genetic interactions between different rice genes were frequently

reported. However, a global view of the interaction networks for all functionally characterized rice genes remains to be elaborated. We constructed interaction networks of functionally characterized genes based on the concurrence of the symbols of two or more genes in the same sentence of an abstract or a title of research publications using in-house R script with manual curation. A sentence, in which two or more genes were observed, was regarded as an evidence supporting the connection between these genes. In total, 1,841 connections supported by 4,046 evidences were detected, which comprised 1,310 genes constituting 214 interaction networks (Supplementary Table S7).

The largest network was composed of 762 genes including ones associated with flowering, phosphate uptake and homeostasis, iron uptake, stress signaling, blight disease resistance, meiosis, BR and GA signaling, grain weight, and endosperm development (Figure 3). Genes related to the same trait were clustered together, indicating the trustworthy of this approach. The enormous size of this network was mainly caused by pleiotropic genes involved in different biological pathways. For example, *Ghd8* was responsible for grain number, plant height and heading date [28]. *Ghd8* connected to genes controlling heading date including *Ehd1* [29], *Hd16* [30], and *RFT1* [29], and genes controlling tillering including *MOC1* [31], which was further connected with *MIP1*, a gene regulating tillering and plant height [32]. The other 213 interaction networks were made up of 548 rice genes, 88% of which contained only two or three genes (Supplementary Figure S5). The second largest network contained 14 genes involved in glutamine metabolism, including *OsAMT1;3*,

GAD3, and *GAT1* [33, 34]. Genes in terms of small RNA biogenesis including *OsDCL3a*, *OsDCL1* and *OsHEN1* were observed in a 10-gene network [35-37] (Supplementary Figure S5).

We further constructed an interaction network using 77 genes involved in flowering regulation (Figure 4). Based on the orthologous groups among seven plants provided by the Rice Genome Annotation Project (http://rice.plantbiology.msu.edu/annotation_pseudo_apk.shtml), we found that 40 of the 77 genes had orthologous genes in sorghum, maize, Brachypodium, Arabidopsis, poplar and grapevine, and orthologous genes were also identified for another 20 rice genes in sorghum, maize and Brachypodium (Figure 4, Supplementary Table S8). Only seven genes, *RFT1*, *Ehd4*, *Hd6*, *OsCO3*, *ROC4*, *Se14* and *OsPIL15*, were unique to rice. These results demonstrated the increasing degree of conservation of the flowering pathway among plants with closer phylogenetic relationships, implying substantial value of knowledge on functionally characterized rice genes to future dissection of flowering time regulation in other crops.

Discussion

In this study, we built a comprehensive and accurate database of functionally characterized rice genes. Along with the sequence and phenotype data of thousands of rice accessions reported in recent years, the affluent information of rice genes in our database would enable further exploring of the crosslink between gene functions and natural variations. We found that a cloned rice gene *OsSGL* (LOC_Os02g04130,

chr02:1799733-1800811), which regulated grain weight in rice, was ~70 kb away from a GWAS peak (chr02:1871732) in terms of grain weight [38, 39]. Likewise, another gene *OsPPKL3* (LOC_Os12g42310, chr12:26273157-26282197), which regulated grain length, was ~90 kb away from a GWAS peak (chr12:26182880) associated with grain length [40, 41]. The functions of *OsSGL* and *OsPPKL3* were characterized by transgenic studies and the natural variations of the two genes are yet to be dissected. Our database is also beneficial to the interpretation of the large scale DNA, mRNA and other sequencing dataset in rice. Analyses of these data usually identify differentially expressed genes, gene co-expression networks, differentially methylated regions and ChIP-seq peaks, etc. The detailed information concerning several thousands of rice genes provided in this database would be helpful for illustration of these results [42]. In addition, our work in rice would facilitate functional genomics studies in other crops including wheat, sorghum, and maize based on homolog information.

Pyramiding and editing of functionally characterized rice genes regulating important agronomic traits by molecular marker assisted selection and CRISPR are two promising approaches used to breeding new rice varieties in recent years [43-45]. Thus, this database would play important roles in future rice breeding. For a specific agronomic trait, all related genes could be retrieved from this database conveniently for further manipulation (https://funricegenes.github.io/tags/#blight_disease). For any of these genes, all relevant publications and a brief summary were available in this database (https://funricegenes.github.io/xa21/). The sequences of different alleles

reported were also archived in this database. These resources would greatly facilitate breeding design to improve target agronomic traits by pyramiding of elite alleles or knocking out deleterious alleles. In addition, the effect of one gene might be enhanced or masked by other genes [46]. Thus, the gene interaction networks provided in this database could also be taken into account when making breeding designs.

Materials and Methods

Geocoding of author affiliations

The latitudes and longitudes of all the author affiliations were obtained using the application interface (API) provided by the DATASCIENCETOOLKIT website (http://www.datasciencetoolkit.org/) with in-house R scripts. For author affiliations failed to be geocoded at high resolutions, we further manually used the Mapeasy website (http://www.mapseasy.com/adress-to-gps-coordinates.php) to find the accurate latitudes and longitudes. The R package ggmap was used to demonstrate the positions of all affiliations on the world map [47].

Extraction of information from PDF files

The occurrence of keywords, including map-based cloning, positional cloning, accession number, accession No., northern blot, northern analysis, northern hybridization and the regular expression "os[0-1][0-9]g[0-9]+.*", in PDF files were inspected utilizing the R tm [48] package.

Construction of interaction networks

The R package igraph [49] were used to build the interaction networks based on all

the connection information between genes. The networks were then exported in format suitable for Cytoscape, which was used to visualize the network [50].

Additional files

Additional file 1: Table S1: A comprehensive list of functionally characterized rice genes.

Additional file 2: Table S2: List of rice gene families.

Additional file 3: Table S3: Publications on functionally characterized rice genes.

Additional file 4: Table S4: The geocoding results of author affiliations.

Additional file 5: Table S5: Genes with different functions that were assigned the same symbol.

Additional file 6: Table S6: Concurrence of the genes symbols and the keywords regarding phenotype description or biological process in the same sentence of abstracts or titles of literatures.

Additional file 7: Table S7: Concurrence of the symbols of two or more genes in the same sentence of abstracts or titles of research publications.

Additional file 8: Table S8: Orthologs of genes regulating heading date in rice.

Additional file 9: Figure S1. Number of papers on rice functional genomics studies published in each year.

Additional file 10: Figure S2: Word cloud analysis of the titles of all the publications on rice functional genomics studies.

Additional file 11: Figure S3: Word cloud analysis of the abstracts of all the

publications on rice functional genomics studies.

Additional file 12: Figure S4: Global distribution of affiliations contributed to rice functional genomics studies. All the affiliations are marked on the world map as blue circles based on their longitude and latitude. The size of the circle represents the number of publications conducted by each affiliation. Data after 18 Jun 2015 are not shown.

Additional file 13: Figure S5: Gene interaction networks constructed based on the concurrence of two or more genes in the same sentence of abstracts or titles of publications. Each white node represents a gene while each green edge indicates a connection between two genes.

Conflicts of interest

The authors declare that they have no competing interests.

Authors' Contributions

W.Y. conceived and designed the experiments; W.Y., G.L., Y.Y. and Y.O. analyzed the data; W.Y. and Y.O. wrote the paper.

Acknowledgements

Not applicable.

Funding

This research was supported by grants from the National Natural Science Foundation of China (31371599 and 31201190), and the Outstanding Young Talents Program.

Reference

1. Fontana L and Partridge L. Promoting Health and Longevity through Diet: From Model Organisms to Humans. Cell. 2015;161 1:106-18. doi:10.1016/j.cell.2015.02.020.
2. Goff SA, Ricke D, Lan TH, Presting G, Wang R, Dunn M, et al. A draft sequence of the rice genome (*Oryza sativa* L. ssp. *japonica*). Science. 2002;296 5565:92-100. doi:10.1126/science.1068275.
3. Wang J, Yu H, Xiong G, Lu Z, Jiao Y, Meng X, et al. Tissue-specific Ubiquitination by IPA1 INTERACTING PROTEIN 1 Modulates IPA1 Protein Levels to Regulate Plant Architecture in Rice. The Plant Cell. 2017; doi:10.1105/tpc.16.00879.
4. Fan C, Xing Y, Mao H, Lu T, Han B, Xu C, et al. *GS3*, a major QTL for grain length and weight and minor QTL for grain width and thickness in rice, encodes a putative transmembrane protein. Theoretical and Applied Genetics. 2006;112 6:1164-71. doi:10.1007/s00122-006-0218-1.
5. Deng Y, Zhai K, Xie Z, Yang D, Zhu X, Liu J, et al. Epigenetic regulation of antagonistic receptors confers rice blast resistance with yield balance. Science. 2017;355 6328:962-5. doi:10.1126/science.aai8898.
6. Gu K, Yang B, Tian D, Wu L, Wang D, Sreekala C, et al. R gene expression induced by a type-III effector triggers disease resistance in rice. Nature. 2005;435 7045:1122-5. doi:10.1038/nature03630.

7. Hu K, Cao J, Zhang J, Xia F, Ke Y, Zhang H, et al. Improvement of multiple agronomic traits by a disease resistance gene via cell wall reinforcement. Nature Plants. 2017;3:17009. doi:10.1038/nplants.2017.9.

8. Zhao Y, Huang J, Wang Z, Jing S, Wang Y, Ouyang Y, et al. Allelic diversity in an NLR gene *BPH9* enables rice to combat planthopper variation. Proceedings of the National Academy of Sciences. 2016;113 45:12850-5. doi:10.1073/pnas.1614862113.

9. Xu K, Xu X, Fukao T, Canlas P, Maghirang-Rodriguez R, Heuer S, et al. *Sub1A* is an ethylene-response-factor-like gene that confers submergence tolerance to rice. Nature. 2006;442 7103:705-8. doi:10.1038/nature04920.

10. Tan J, Tan Z, Wu F, Sheng P, Heng Y, Wang X, et al. A Novel Chloroplast-Localized Pentatricopeptide Repeat Protein Involved in Splicing Affects Chloroplast Development and Abiotic Stress Response in Rice. Molecular plant. 2014;7 8:1329-49. doi:10.1093/mp/ssu054.

11. Jiang H, Feng Y, Bao L, Li X, Gao G, Zhang Q, et al. Improving blast resistance of Jin 23B and its hybrid rice by marker-assisted gene pyramiding. Molecular Breeding. 2012;30 4:1679-88. doi:10.1007/s11032-012-9751-6.

12. Wang S, Wu K, Yuan Q, Liu X, Liu Z, Lin X, et al. Control of grain size, shape and quality by *OsSPL16* in rice. Nat Genet. 2012;44 8:950-4. doi:10.1038/ng.2327.

13. Shan Q, Zhang Y, Chen K, Zhang K and Gao C. Creation of fragrant rice by targeted knockout of the *OsBADH2* gene using TALEN technology. Plant

Biotechnology Journal. 2015;13 6:791-800. doi:10.1111/pbi.12312.

14. Bednarek J, Boulaflous A, Girousse C, Ravel C, Tassy C, Barret P, et al. Down-regulation of the *TaGW2* gene by RNA interference results in decreased grain size and weight in wheat. Journal of Experimental Botany. 2012;63 16:5945-55. doi:10.1093/jxb/ers249.

15. LIU Ya-Nan XX-C, HE Zhong-Hu. Characterization of Dense and Erect Panicle 1 Gene (*TaDep1*) Located on Common Wheat Group 5 Chromosomes and Development of Allele-Specific Markers. Acta Agron Sin. 2013;39 04:589-98. doi:10.3724/sp.j.1006.2013.00589.

16. Nemoto Y, Kisaka M, Fuse T, Yano M and Ogihara Y. Characterization and functional analysis of three wheat genes with homology to the CONSTANS flowering time gene in transgenic rice. The Plant Journal. 2003;36 1:82-93. doi:10.1046/j.1365-313X.2003.01859.x.

17. Nakamura S, Abe F, Kawahigashi H, Nakazono K, Tagiri A, Matsumoto T, et al. A Wheat Homolog of MOTHER OF FT AND TFL1 Acts in the Regulation of Germination. The Plant Cell. 2011;23 9:3215-29. doi:10.1105/tpc.111.088492.

18. Comadran J, Kilian B, Russell J, Ramsay L, Stein N, Ganal M, et al. Natural variation in a homolog of Antirrhinum CENTRORADIALIS contributed to spring growth habit and environmental adaptation in cultivated barley. Nat Genet. 2012;44 12:1388-92. doi:10.1038/ng.2447.

19. Yang Q, Li Z, Li W, Ku L, Wang C, Ye J, et al. CACTA-like transposable

element in *ZmCCT* attenuated photoperiod sensitivity and accelerated the postdomestication spread of maize. Proceedings of the National Academy of Sciences. 2013;110 42:16969-74. doi:10.1073/pnas.1310949110.

20. Lamesch P, Berardini TZ, Li D, Swarbreck D, Wilks C, Sasidharan R, et al. The Arabidopsis Information Resource (TAIR): improved gene annotation and new tools. Nucleic acids research. 2012;40 Database issue:D1202-10. doi:10.1093/nar/gkr1090.

21. Gramates LS, Marygold SJ, Santos Gd, Urbano J-M, Antonazzo G, Matthews BB, et al. FlyBase at 25: looking to the future. Nucleic acids research. 2017;45 D1:D663-D71. doi:10.1093/nar/gkw1016.

22. Kawahara Y, de la Bastide M, Hamilton J, Kanamori H, McCombie W, Ouyang S, et al. Improvement of the Oryza sativa Nipponbare reference genome using next generation sequence and optical map data. Rice. 2013;6 1:1-10. doi:10.1186/1939-8433-6-4.

23. Sakai H, Lee SS, Tanaka T, Numa H, Kim J, Kawahara Y, et al. Rice Annotation Project Database (RAP-DB): An Integrative and Interactive Database for Rice Genomics. Plant and Cell Physiology. 2013;54 2:e6. doi:10.1093/pcp/pcs183.

24. Zhang Q, Li J, Xue Y, Han B and Deng XW. Rice 2020: A Call For An International Coordinated Effort In Rice Functional Genomics. Molecular plant. 2008;1 5:715-9. doi:10.1093/mp/ssn043.

25. Zhang J, Chen L-L, Xing F, Kudrna DA, Yao W, Copetti D, et al. Extensive

sequence divergence between the reference genomes of two elite indica rice varieties Zhenshan 97 and Minghui 63. Proceedings of the National Academy of Sciences. 2016; doi:10.1073/pnas.1611012113.

26. Si L, Chen J, Huang X, Gong H, Luo J, Hou Q, et al. *OsSPL13* controls grain size in cultivated rice. Nat Genet. 2016;48 4:447-56. doi:10.1038/ng.3518.

27. Yamauchi T, Yoshioka M, Fukazawa A, Mori H, Nishizawa NK, Tsutsumi N, et al. An NADPH Oxidase RBOH Functions in Rice Roots during Lysigenous Aerenchyma Formation under Oxygen-Deficient Conditions. The Plant Cell. 2017; doi:10.1105/tpc.16.00976.

28. Yan WH, Wang P, Chen HX, Zhou HJ, Li QP, Wang CR, et al. A major QTL, *Ghd8*, plays pleiotropic roles in regulating grain productivity, plant height, and heading date in rice. Molecular plant. 2011;4 2:319-30. doi:10.1093/mp/ssq070.

29. Dai X, Ding Y, Tan L, Fu Y, Liu F, Zhu Z, et al. *LHD1*, an Allele of *DTH8/Ghd8*, Controls Late Heading Date in Common Wild Rice (*Oryza rufipogon*)F. Journal of Integrative Plant Biology. 2012;54 10:790-9. doi:10.1111/j.1744-7909.2012.01166.x.

30. Hori K, Ogiso-Tanaka E, Matsubara K, Yamanouchi U, Ebana K and Yano M. *Hd16*, a gene for casein kinase I, is involved in the control of rice flowering time by modulating the day-length response. The Plant Journal. 2013;76 1:36-46. doi:10.1111/tpj.12268.

31. Li X, Qian Q, Fu Z, Wang Y, Xiong G, Zeng D, et al. Control of tillering in

rice. Nature. 2003;422 6932:618-21. doi:10.1038/nature01518.

32. Sun F, Zhang W, Xiong G, Yan M, Qian Q, Li J, et al. Identification and functional analysis of the *MOC1* interacting protein 1. Journal of Genetics and Genomics. 2010;37 1:69-77. doi:10.1016/s1673-8527(09)60026-6.

33. Yang S, Hao D, Cong Y, Jin M and Su Y. The rice *OsAMT1;1* is a proton-independent feedback regulated ammonium transporter. Plant Cell Reports. 2015;34 2:321-30. doi:10.1007/s00299-014-1709-1.

34. El-kereamy A, Bi Y-M, Ranathunge K, Beatty PH, Good AG and Rothstein SJ. The Rice R2R3-MYB Transcription Factor *OsMYB55* Is Involved in the Tolerance to High Temperature and Modulates Amino Acid Metabolism. PLOS ONE. 2012;7 12:e52030. doi:10.1371/journal.pone.0052030.

35. Wei L, Gu L, Song X, Cui X, Lu Z, Zhou M, et al. Dicer-like 3 produces transposable element-associated 24-nt siRNAs that control agricultural traits in rice. Proceedings of the National Academy of Sciences. 2014;111 10:3877-82. doi:10.1073/pnas.1318131111.

36. Liu B, Li P, Li X, Liu C, Cao S, Chu C, et al. Loss of Function of *OsDCL1* Affects MicroRNA Accumulation and Causes Developmental Defects in Rice. Plant Physiology. 2005;139 1:296-305. doi:10.1104/pp.105.063420.

37. Abe M, Yoshikawa T, Nosaka M, Sakakibara H, Sato Y, Nagato Y, et al. WAVY LEAF1, an Ortholog of Arabidopsis HEN1, Regulates Shoot Development by Maintaining MicroRNA and Trans-Acting Small Interfering RNA Accumulation in Rice. Plant Physiology. 2010;154 3:1335-46.

doi:10.1104/pp.110.160234.

38. Wang M, Lu X, Xu G, Yin X, Cui Y, Huang L, et al. *OsSGL*, a novel pleiotropic stress-related gene enhances grain length and yield in rice. Scientific Reports. 2016;6:38157. doi:10.1038/srep38157.

39. Yang W, Guo Z, Huang C, Duan L, Chen G, Jiang N, et al. Combining high-throughput phenotyping and genome-wide association studies to reveal natural genetic variation in rice. Nature Communications. 2014;5:5087. doi:10.1038/ncomms6087.

40. Zhang X, Wang J, Huang J, Lan H, Wang C, Yin C, et al. Rare allele of *OsPPKL1* associated with grain length causes extra-large grain and a significant yield increase in rice. Proceedings of the National Academy of Sciences. 2012;109 52:21534-9. doi:10.1073/pnas.1219776110.

41. McCouch SR, Wright MH, Tung C-W, Maron LG, McNally KL, Fitzgerald M, et al. Open access resources for genome-wide association mapping in rice. Nature Communications. 2016;7:10532. doi:10.1038/ncomms10532.

42. Zong W, Tang N, Yang J, Peng L, Ma S, Xu Y, et al. Feedback regulation of ABA signaling and biosynthesis by a bZIP transcription factor targets drought resistance related genes. Plant Physiology. 2016; doi:10.1104/pp.16.00469.

43. Collard BC and Mackill DJ. Marker-assisted selection: an approach for precision plant breeding in the twenty-first century. Philosophical Transactions of the Royal Society B: Biological Sciences. 2008;363 1491:557-72. doi:10.1098/rstb.2007.2170.

44. Zeng D, Tian Z, Rao Y, Dong G, Yang Y, Huang L, et al. Rational design of high-yield and superior-quality rice. Nature Plants. 2017;3:17031. doi:10.1038/nplants.2017.31.

45. Zhou H, He M, Li J, Chen L, Huang Z, Zheng S, et al. Development of Commercial Thermo-sensitive Genic Male Sterile Rice Accelerates Hybrid Rice Breeding Using the CRISPR/Cas9-mediated *TMS5* Editing System. Scientific Reports. 2016;6:37395. doi:10.1038/srep37395.

46. Gao X, Zhang X, Lan H, Huang J, Wang J and Zhang H. The additive effects of *GS3* and *qGL3* on rice grain length regulation revealed by genetic and transcriptome comparisons. BMC Plant Biology. 2015;15 1:156. doi:10.1186/s12870-015-0515-4.

47. Kahle D and Wickham H. ggmap: Spatial Visualization with ggplot2. The R Journal. 2013;5 1:144-61.

48. Meyer D, Hornik K and Feinerer I. Text mining infrastructure in R. Journal of Statistical Software. 2008;25 5:1-54.

49. Csardi G and Nepusz T. The igraph Software Package for Complex Network Research. InterJournal. 2006;Complex Systems:1695. doi:citeulike-article-id:3443126.

50. Shannon P, Markiel A, Ozier O, Baliga NS, Wang JT, Ramage D, et al. Cytoscape: A Software Environment for Integrated Models of Biomolecular Interaction Networks. Genome Research. 2003;13 11:2498-504. doi:10.1101/gr.1239303.

Figure legends

Figure 1. Chromosome distribution of representative functionally characterized rice genes.

The chromosomes are represented as vertical rectangles and each horizontal line on it denotes the position of a functionally characterized rice gene. Symbols of all genes are labeled. A total of 930 representative genes are shown.

Figure 2. Usage of various biotechniques in rice functional genomics studies.

The *y*-axis indicates the number of publications using a specific biotechnique. Data after 18 Jun 2015 are not shown.

Figure 3. The gene interaction network comprising 762 genes.

Each white node represents a functionally characterized rice gene and gene symbols are marked beside each node. Each green edge indicates a connection between two genes. Genes involved in the same biological pathways are indicated.

Figure 4. Interaction network of genes regulating flowering in rice and the orthologs of these genes in other plants.

Each node represents a functionally characterized rice gene. Each edge indicates a connection between two genes. Genes with different number of orthologous genes were indicated with different color and shape. “Rice + (Maize | Poplar)” indicates “Rice and Maize” or “Rice and Poplar”. Detailed information are shown in Supplementary Table S8.

Figure 1

Click here to download Figure Figure_1.pdf

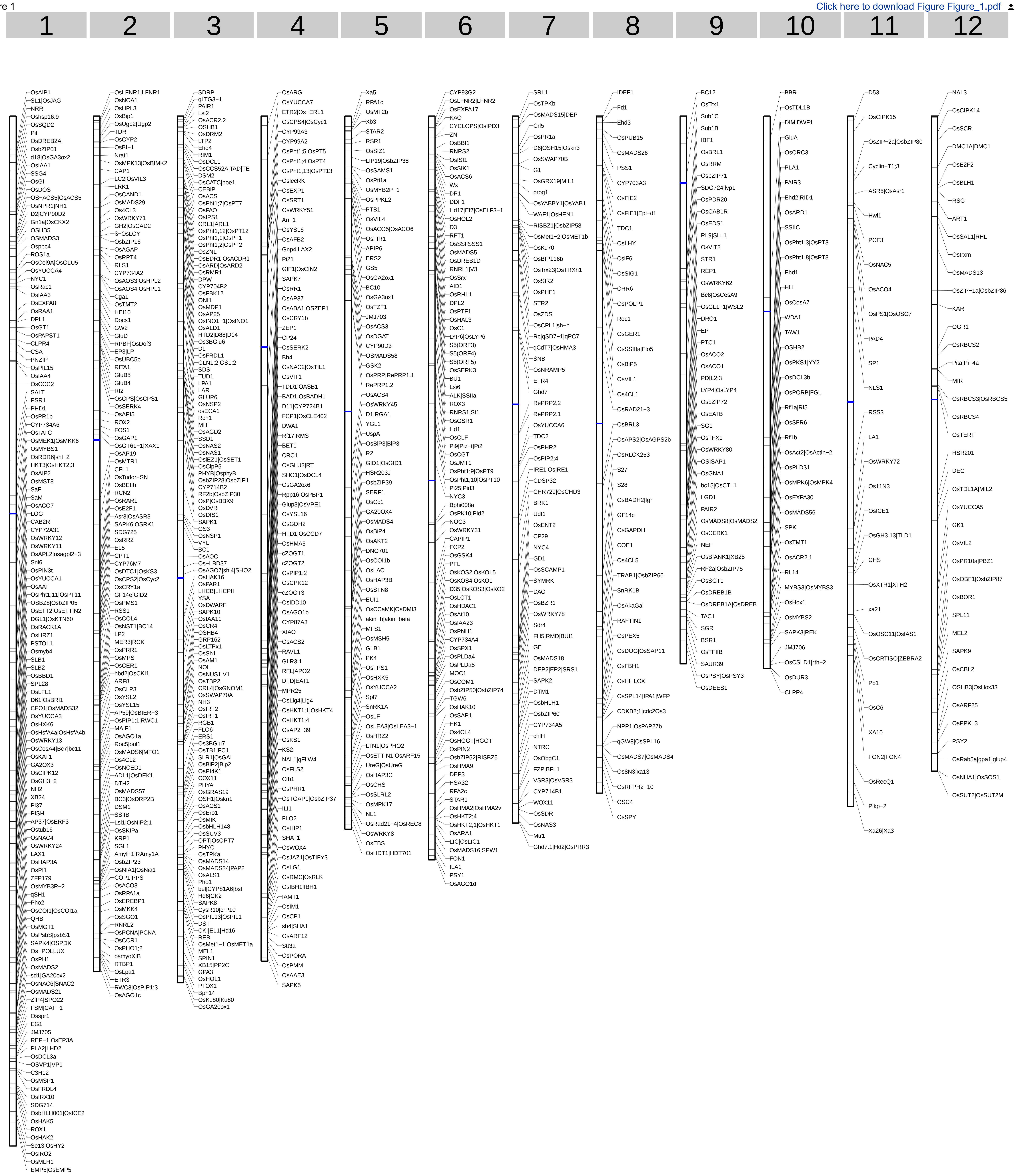

Figure 2

Click here to download Figure Figure_2.pdf

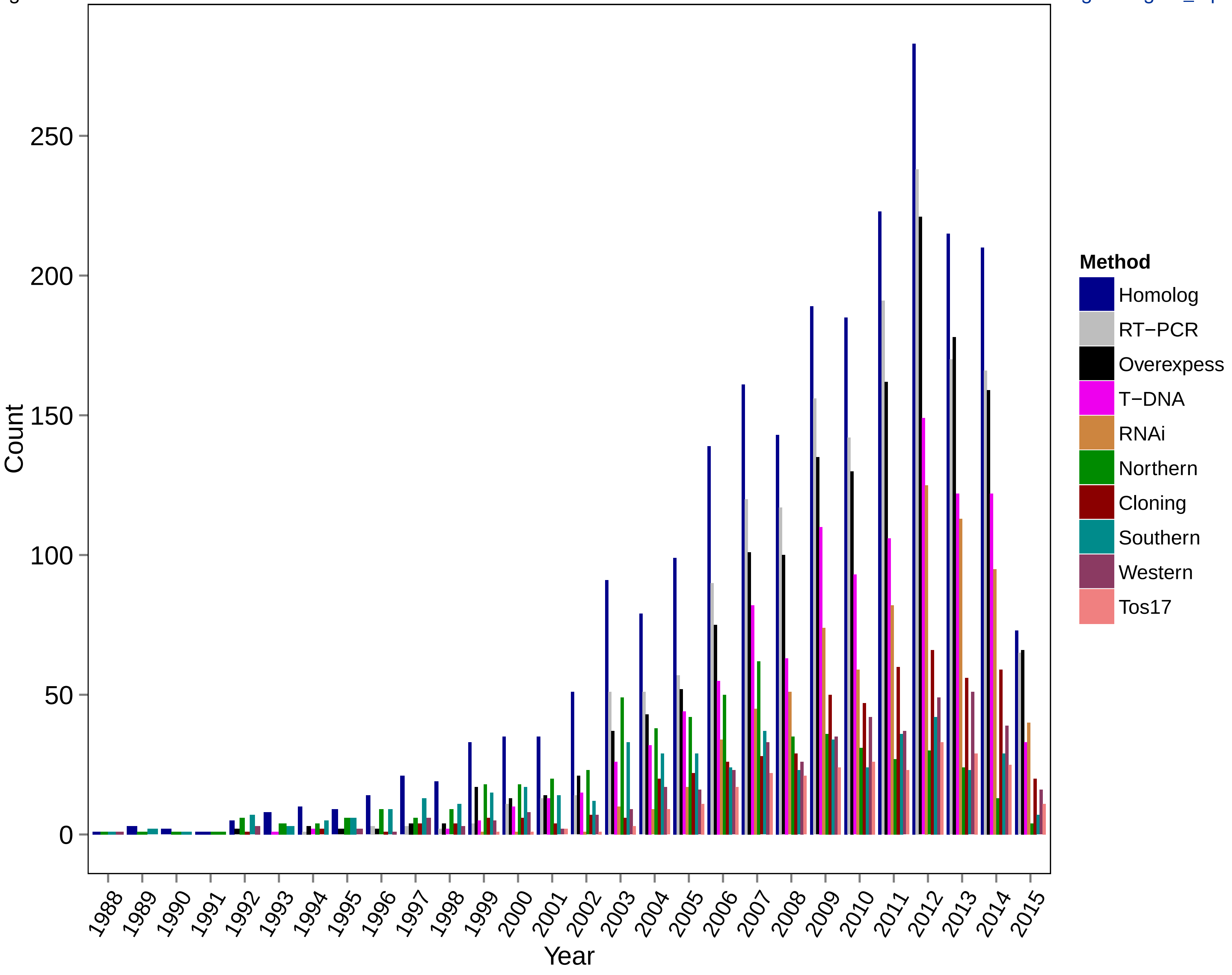

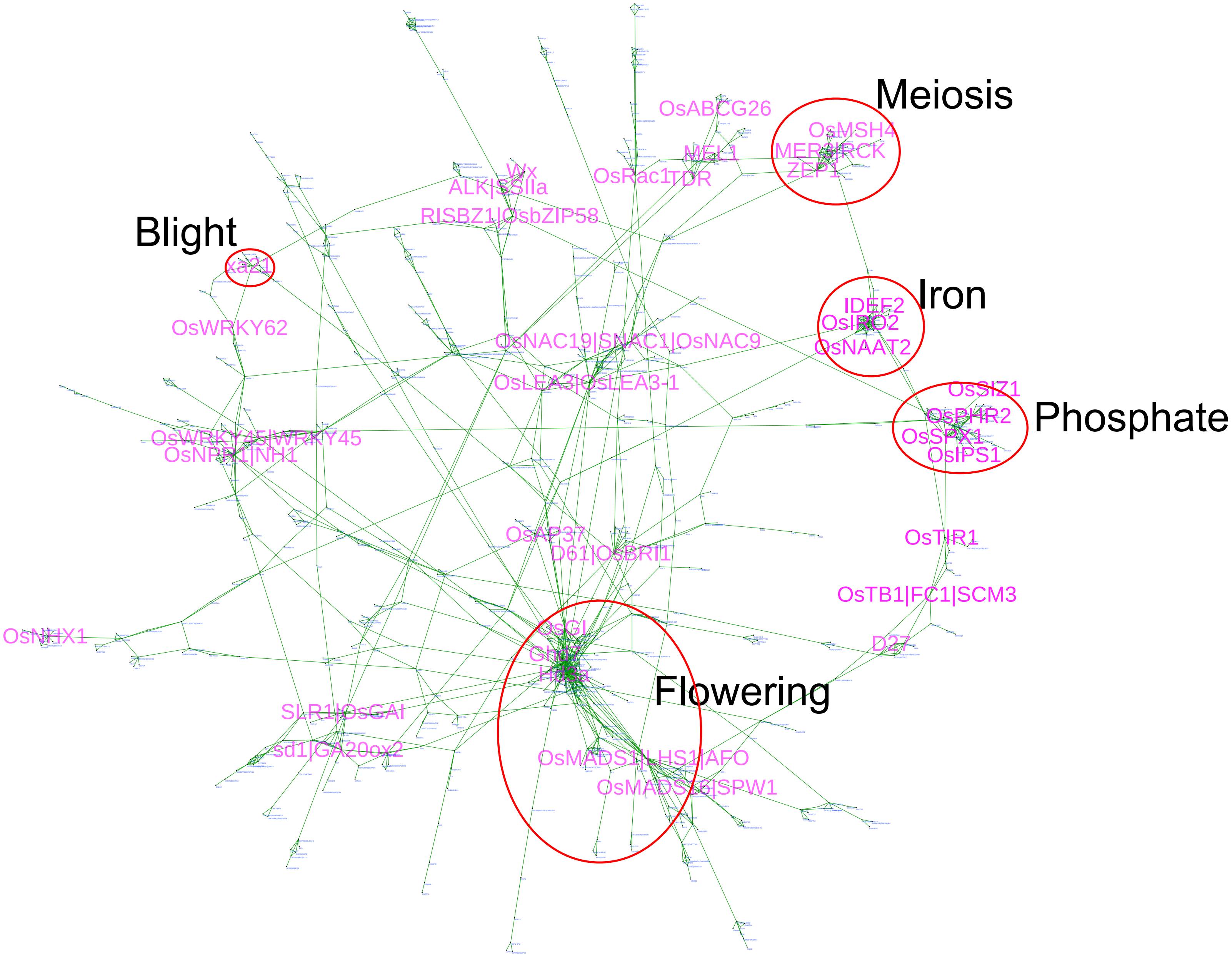
Meiosis
OsABCG26
OsMSH4
MEL1
ZEP1
OsRac1
TDR
Wx
ALK|SSIIa
RISBZ1|OsbZIP58
Blight
xa21
OsWRKY62
Iron
IDEF2
OsIRO2
OsNAAT2
OsNAC19|SNAC1|OsNAC9
OsLEA3|OsLEA3-1
OsSIZ1
Phosphate
OsPHR2
OsSPX1
OsIPS1
OsWRKY45|WRKY45
OsNPR1|NH1
OsAP37
D61|OsBRI1
OsTIR1
OsTB1|FC1|SCM3
OsNHX1
D27
OsGI
Flowering
SLR1|OsGAI
sd1|GA20ox2
OsMADS1|LHS1|AFO
OsMADS16|SPW1

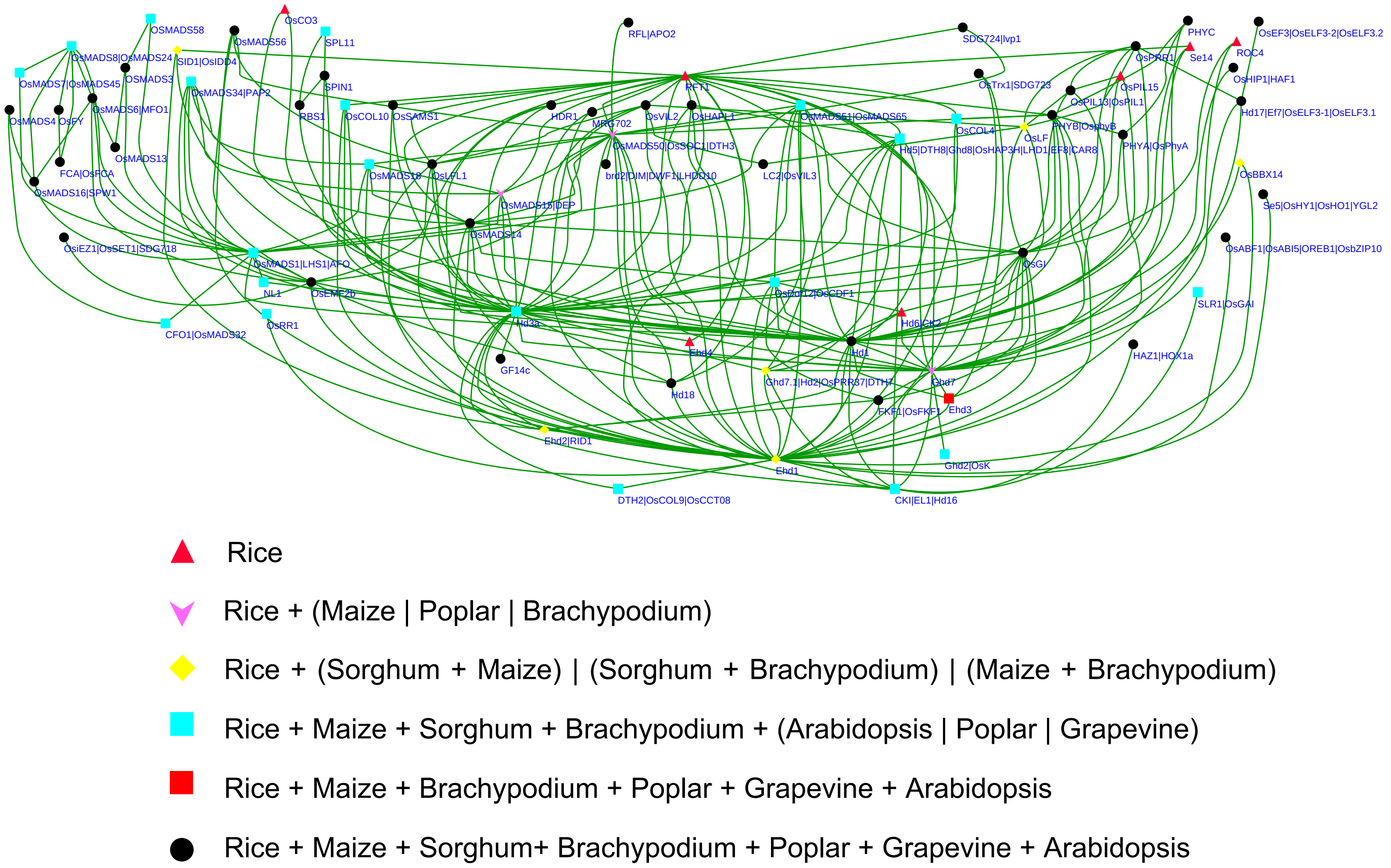

OsCO3
OSMADS58
SPL11
OsMADS56
RFL|APO2
SDG724|lvp1
PHYC
OsEF3|OsELF3-2|OsELF3.2
OsMADS8|OsMADS24
SID1|OsIDD4
OsPRR1
Se14
ROC4
OsMADS7|OsMADS45
OSMADS3
SPIN1
RFT1
OsTrx1|SDG723
OsPIL15
OsHIP1|HAF1
OsMADS34|PAP2
OsPIL13|OsPIL1
Hd17|Ef7|OsELF3-1|OsELF3.1
OsMADS6|MFO1
RBS1
OsCOL10
OsSAMS1
HDR1
MRG702
OsVIL2
OsHAPL1
OsMADS51|OsMADS65
OsMADS4
OsFY
OsCOL4
OsLF
PHYB|OsphyB
OsMADS50|OsSOC1|DTH3
Hd5|DTH8|Ghd8|OsHAP3H|LHD1|EF8|CAR8
PHYA|OsPhyA
OsMADS13
FCA|OsFCA
OsMADS18
OsLFL1
brd2|DIM|DWF1|LHDD10
LC2|OsVIL3
OsBBX14
OsMADS16|SPW1
OsMADS15|DEP
Se5|OsHY1|OsHO1|YGL2
OsMADS14
OsiEZ1|OsSET1|SDG718
OsMADS1|LHS1|AFO
OsGI
OsABF1|OsABI5|OREB1|OsbZIP10
NL1
OsEMF2b
OsDof12|OsCDF1
SLR1|OsGAI
Hd3a
Hd6|CK2
OsRR1
CFO1|OsMADS32
Ehd4
Hd1
GF14c
HAZ1|HOX1a
Ghd7.1|Hd2|OsPRR37|DTH7
Ghd7
Hd18
FKF1|OsFKF1
Ehd3
Ehd2|RID1
Ghd2|OsK
Ehd1
DTH2|OsCOL9|OsCCT08
CKI|EL1|Hd16
Rice
Rice + (Maize | Poplar | Brachypodium)
Rice + (Sorghum + Maize) | (Sorghum + Brachypodium) | (Maize + Brachypodium)
Rice + Maize + Sorghum + Brachypodium + (Arabidopsis | Poplar | Grapevine)
Rice + Maize + Brachypodium + Poplar + Grapevine + Arabidopsis
Rice + Maize + Sorghum+ Brachypodium + Poplar + Grapevine + Arabidopsis

Figure_S1

Click here to access/download
Supplementary Material
Supplementary_Figure_S1.tif

Figure_S2

Click here to access/download
Supplementary Material
Supplementary_Figure_S2.tif

Figure_S3

Click here to access/download
Supplementary Material
Supplementary_Figure_S3.tif

Figure_S4

Click here to access/download
Supplementary Material
Supplementary_Figure_S4.tif

Figure_S5

Click here to access/download
Supplementary Material
Supplementary_Figure_S5.tif

Table_S1

Click here to access/download
Supplementary Material
Supplementary_Table_S1.xlsx

Table_S2

Click here to access/download
Supplementary Material
Supplementary_Table_S2.xlsx

Table_S3

Click here to access/download
Supplementary Material
Supplementary_Table_S3.xlsx

Table_S4

Click here to access/download
Supplementary Material
Supplementary_Table_S4.xlsx

Table_S5

Click here to access/download
Supplementary Material
Supplementary_Table_S5.xlsx

Table_S6

Click here to access/download
Supplementary Material
Supplementary_Table_S6.xlsx

Table_S7

Click here to access/download
Supplementary Material
Supplementary_Table_S7.xlsx

Table_S8

Click here to access/download
Supplementary Material
Supplementary_Table_S8.xlsx
