# Supplementary figures and images for: funRiceGenes dataset for comprehensive understanding and application of rice functional genes
Source: Gigascience. 2017 Dec 4;7(1):gix119. doi: 10.1093/gigascience/gix119 (PMC5765555; doi:10.1093/gigascience/gix119)

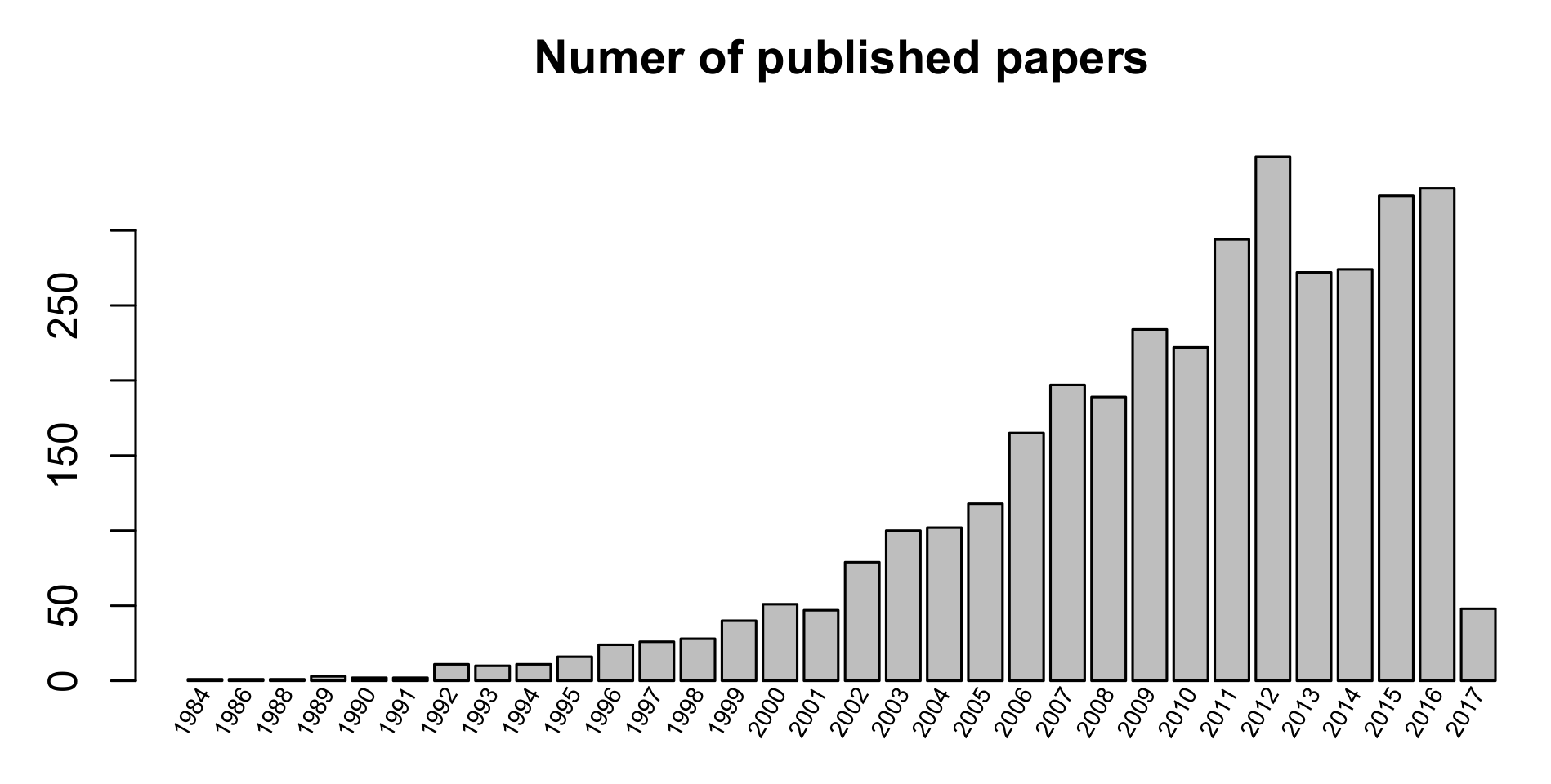

Supplement: Supplement materials [file gix119_supp.zip › Figure_S1.tif]

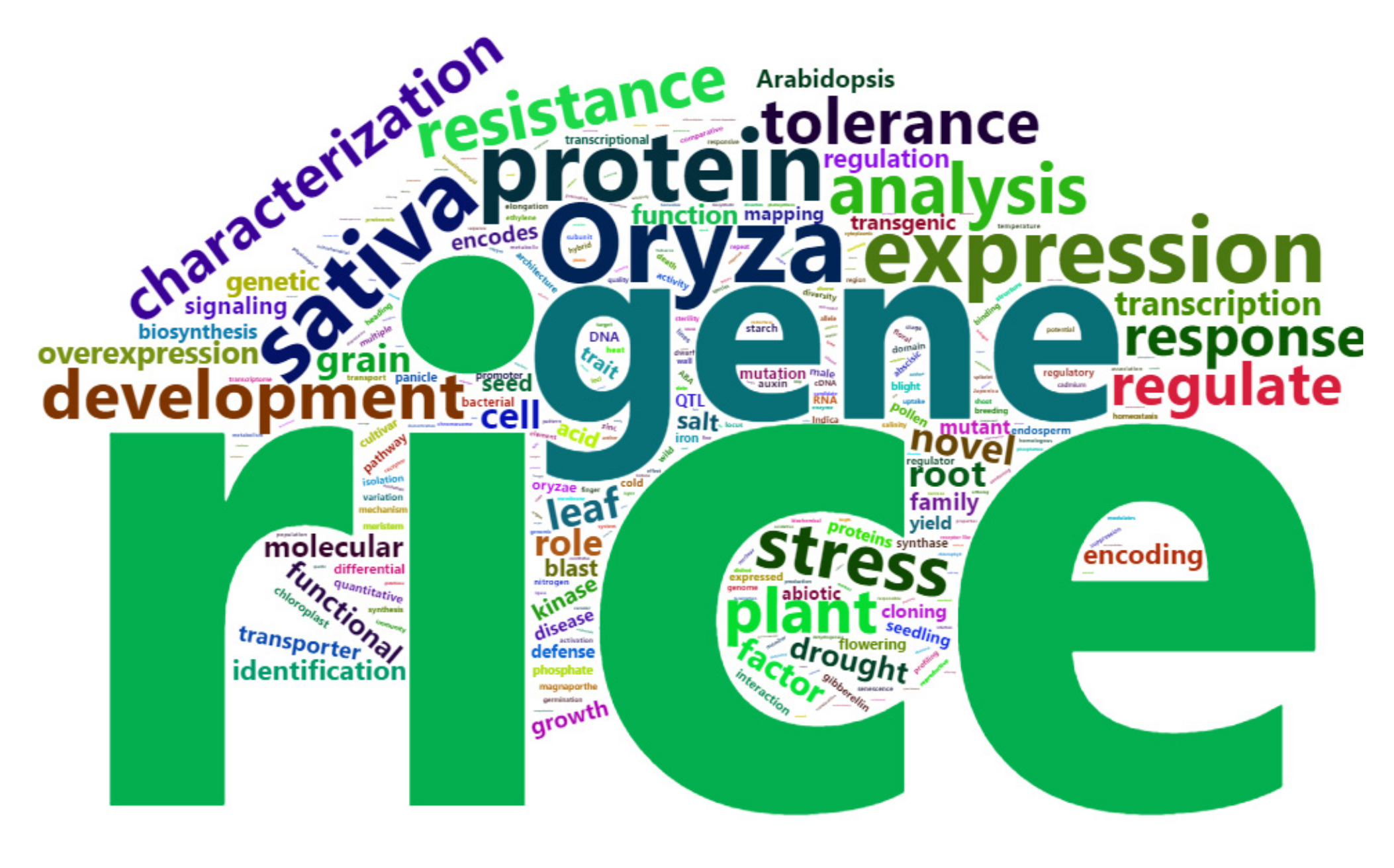

Supplement: Supplement materials [file gix119_supp.zip › Figure_S2.tif]

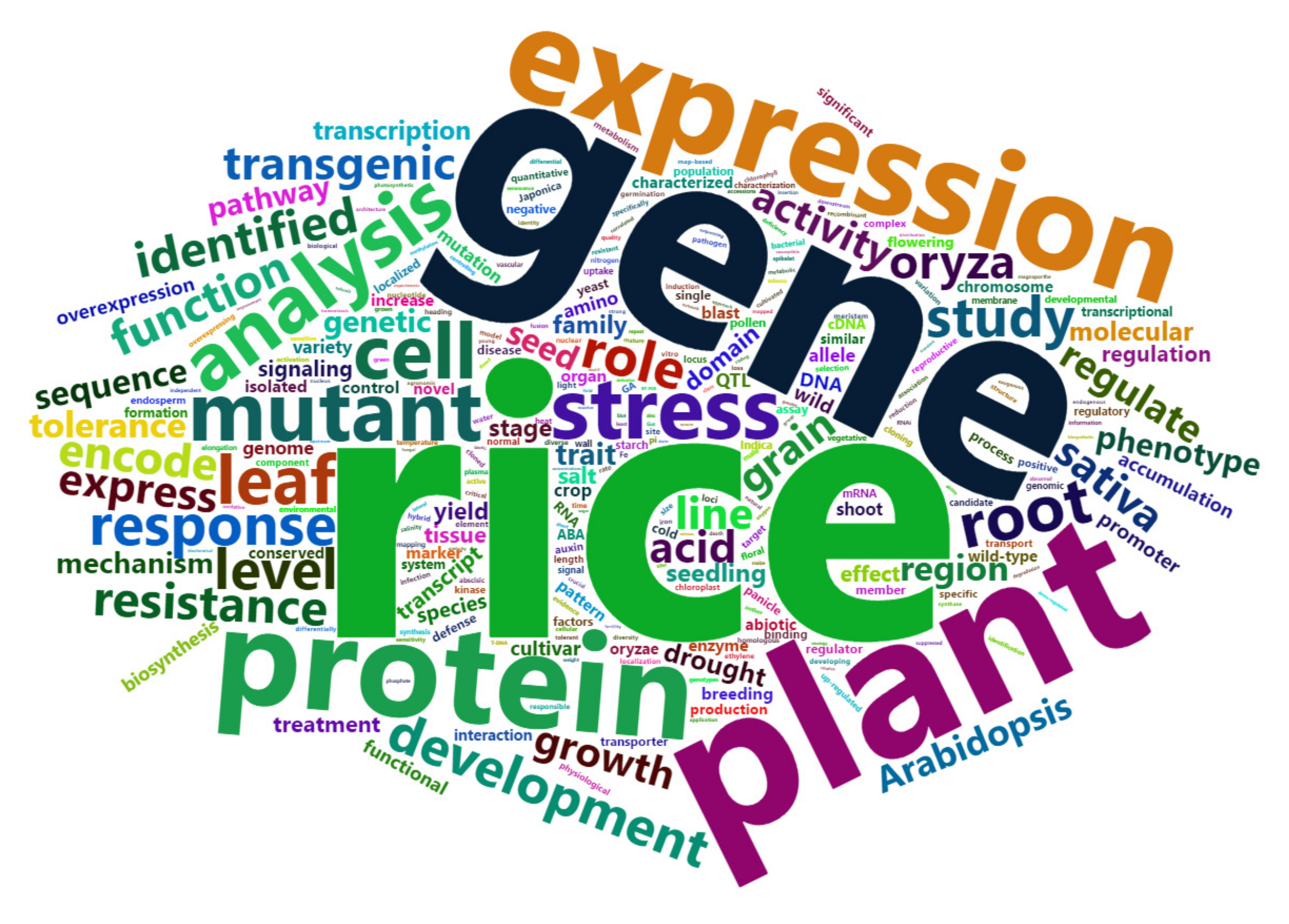

Supplement: Supplement materials [file gix119_supp.zip › Figure_S3.tif]

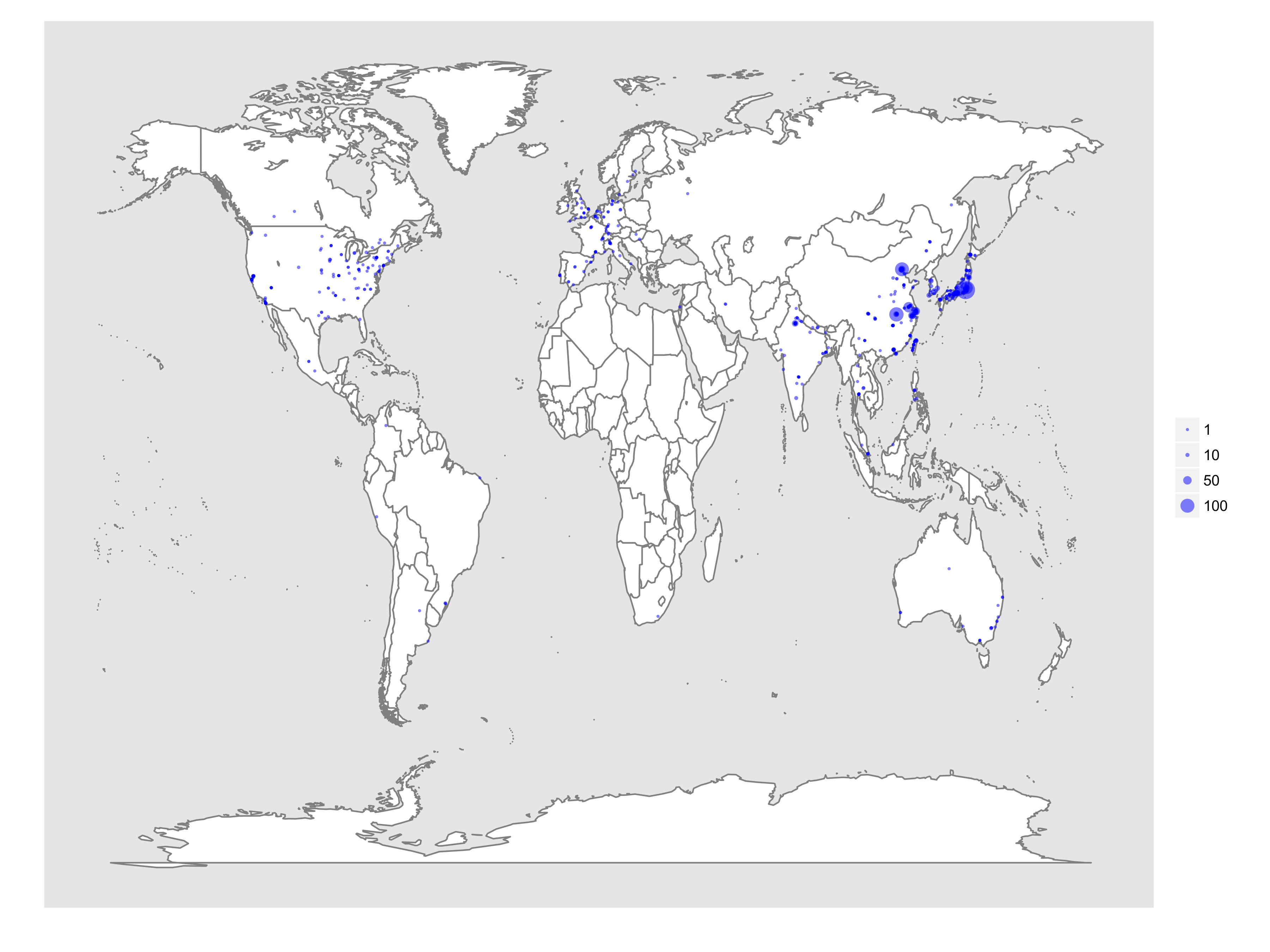

Supplement: Supplement materials [file gix119_supp.zip › Figure_S4.tif]

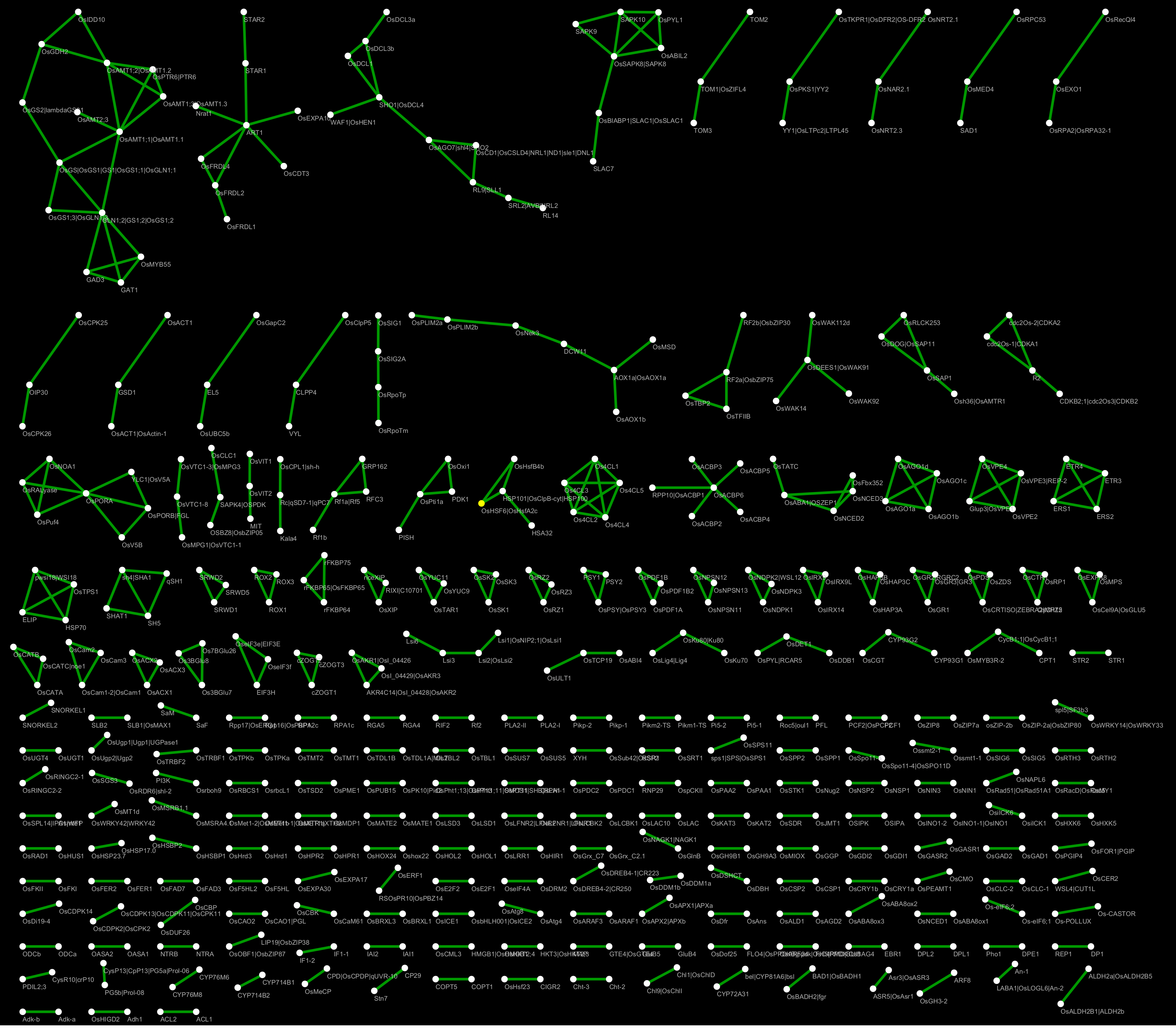

Supplement: Supplement materials [file gix119_supp.zip › Figure_S5.tif]
