# Supplementary material for: funRiceGenes dataset for comprehensive understanding and application of rice functional genes
Source: Gigascience. 2017 Dec 4;7(1):gix119. doi: 10.1093/gigascience/gix119 (PMC5765555; doi:10.1093/gigascience/gix119)
Supplement: Response_to_Reviewer_Comments_Original_Submission.pdf [file gix119_response_to_reviewer_comments_original_submission.pdf]

Author's Response To Reviewer Comments

Dear Editor:

We are now submitting our revised manuscript, entitled funRiceGenes dataset for comprehensive understanding and application of rice functional genes, to your consideration for publication in GigaScience. This manuscript was assigned GIGA-D-17-00154 in the previous submission. We are grateful to the editor's and reviewers' comments and suggestions.
We have done a thorough revision of the manuscript to address the editor's and reviewers' concerns. We have rewritten many parts of the manuscript to improve the writing of the manuscript. In pages that follow, point-by-point responses to the comments and suggestions by the editor and reviewers are provided.
We will greatly appreciate your attention to this submission.

Sincerely,
Wen Yao, Ph. D.
National Key Laboratory of Crop Genetic Improvement
Huazhong Agricultural University
Wuhan 430070, China

Yidan Ouyang, Ph. D.
Professor, College of Life Science and Technology
National Key Laboratory of Crop Genetic Improvement
Huazhong Agricultural University
Wuhan 430070, China

EDITOR COMMENTS
I agree with reviewer 2 that such a database needs to be up-to-date if we understand correctly, data incorporation was up to 2014 only, and I agree with reviewer 2 that it should be up-to-date.
Response: Thanks for the suggestion and we agree with this. Actually, our database is almost always up-to-date. Data collection from different sources until 2014 mentioned in the manuscript were performed for initial construction of the database. Since then, this database was updated by tracking publications from PubMed and new records from the China Rice Data Center and Oryzabase database using a Shiny application. All the updated records are available at https://funricegenes.github.io/news/. The latest update was performed on Sep 20th, 2017.

In addition, it is not quite clear in how far this new dataset is an advance over existing resources - this needs to be discussed and explained in detail. As reviewer 2 says, "clear examples where functional descriptions were improved by the authors' effort need to be provided.
Response: Many thanks for your valuable suggestions. We discussed the advance of the funRiceGenes database in the first paragraph of the Discussion section of the revised manuscript. In this study, we built a comprehensive and accurate database of functionally characterized rice genes, funRiceGenes, which provides a valuable resource for rice functional genomic studies. funRiceGenes was constructed by integrating data from PubMed, Oryzabase, and China Rice Data Center, and was updated every two weeks using a Shiny application. For each gene in the funRiceGenes database, the gene symbol, the genomic locus in the reference genome and the

published papers on this gene were identified. Compared with Textpresso for Oryza sativa (http://map.lab.nig.ac.jp:8095/textpresso/index.html), which is a comprehensive collection of literatures on rice, we further built the associations between genomic locus or symbol of genes and literatures. Based on the literatures identified for each gene, we summarized the brief functions of each gene and constructed interaction networks for all genes. The evidences supporting the functions of all collected genes and the interaction networks are unique to the funRiceGenes database. In addition, user-friendly query interface and tidy data for downloading are provided in the funRiceGenes database.

An interesting feature of your submission are the automatic updates to the database. Please elaborate on this feature, and how the updates are implemented in practice, as it seems to be a useful functionality that may convince the reviewers regarding the merits of your manuscript.
Response: Many thanks for your valuable suggestions. We have given an in-depth description on the automatic updates to the database (from page 5 line 17-25 to page 6 line 1-4 of the revised manuscript). The process for implementation of the updates using the Shiny application was described in the help manual (https://funricegenes.github.io/help.pdf).
New genes were added to this database using the Shiny application, based on daily email alert of the searching results from the PubMed database with the keyword rice (rice[Title] OR rice[Title/Abstract]) (https://funricegenes.github.io/help.pdf). For all the PubMed records in the email alert, we identified ones on functionally characterized rice genes. We then went over the full publication of each record and identified the gene symbol and gene model in the reference genome. After inputting the gene symbol, the gene model in the reference genome and the PubMed identifier, the Shiny application will fetch the corresponding publication record from PubMed and extract key information automatically. We also kept track of new records in the database of Oryzabase and China Rice Data Center, which were then added to our database using the Shiny application. Since 13 Feb 2014, funRiceGenes was updated every two weeks using the Shiny application. All the updated records are available at https://funricegenes.github.io/news/.

Regarding the article type, in case of acceptance, we feel the manuscript would be suitable as a "Data Note" (https://academic.oup.com/gigascience/pages/data_note), or maybe also as a "Technical Note" - we can discuss this further when you submit a revised manuscript.
Response: Many thanks for your suggestion. We would like to change our manuscript as a "Technical Note".

REVIEWER COMMENTS
Reviewer: 1
The manuscript provides an integration of publicly available information on rice gene functions and associated attributes from heterogenous sources, in order to make the information available for biological interpretation. A number of search tools have been developed or applied to derive associations between heterogeneous data subjects. These associations have also been used to derive networks of functional associations from literature that can provide a basis for further searches.
The interactive search page with a Shiny application for updating was tested with a number of genes of interest, and they made links between loci numbers and new publications, providing a potential gene function from available literature. I see that as a very good tool to test data and hypotheses in a research. Although the interactive page is a bit slow, and might be even more

with more traffic from searches, it is user friendly and would be an asset for researchers doing GWAS or gene function identification. The utility for gene function information goes beyond Gramene and RAPdb, but will only be able to remain so if the planned automatic updates to the database remain functional.
Response: Many thanks for the positive comments. We updated the funRiceGenes database every two weeks since its initial construction in 2014. Since 2014, this database was updated using a Shiny application by tracking publications from PubMed and new records in the China Rice Data Center and Oryzabase databases. All updated records are available at https://funricegenes.github.io/news/, with the latest update performed on Sep 20th, 2017. We will keep updating of the funRiceGenes database in future.
The speed of the interactive page is probably restricted by the internet speed in our university. However, the Shiny application can be downloaded and deployed on local computer, which can be then accessed without speed limit. Please check the help manual (https://funricegenes.github.io/help.pdf) for downloading and deploying of the Shiny application on local computer.

Since the Nipponbare genome basis and annotation is used, is there a potential to survey overlapping genomic intervals from the indica genome sequences and make predictions of intervening syntenic genes?
Response: Thanks for your valuable suggestion. We provide functions allowing conversion between indica and japonica syntenic gene IDs in the IDConversion menu of the updated Shiny application (http://funricegenes.ncpgr.cn/), based on synteny analysis between Nipponbare genome and two high-quality indica reference genomes reported in Zhang et al. 2016, PNAS (http://www.pnas.org/content/113/35/E5163.full). In the conversion result, we provide links to the RIGW database (http://rice.hzau.edu.cn/), which contains the detailed information for the indica genes. In the RIGW database, syntenic alignments between the Nipponbare and two indica genomes are provided (http://rice.hzau.edu.cn/cgi-bin/gb2/gbrowse_syn/3rice_syn/).

Is the search scalable to use larger datasets or gene lists rather than individual genes to derive hypotheses from experimental data, eg what would be the pathways affected from mutation of a specific candidate gene, when no experimental data is available? Or, could one predict candidate genes that might perturb/affect a specific biological process. The availability of other network-based predictive methods and integration into funRiceGenes would be able to provide further tools for experimenters.
Response: Thanks for your valuable suggestions. We provided batch query functions allowing search of the funRiceGenes database with gene lists in the Download menu of the updated Shiny application (http://funricegenes.ncpgr.cn/). We also integrated the data from the RiceNet V2 database into funRiceGenes, which provides genome-scale probabilistic functional gene networks of O. sativa (RiceNet v2: an improved network prioritization server for rice genes, Nucl. Acids Res, 2015, 43:W122-7).

The funRiceGenes application on publications has similarities to the Textpresso application for many model systems from Arabidopsis (http://www.textpresso.org/arabidopsis/) to mouse and also initiated for Oryza sativa (http://map.lab.nig.ac.jp:8095/textpresso/index.html). This rice functional genomics application funRiceGenes should be shown how it distinguishes from the textpresso tool with differences outlined in the manuscript.

Response: Many thanks for your valuable suggestions. Textpresso provides an archive of biological literature allowing information extracting by keywords. Only if the symbol of a gene is present in the title and/or the abstract of published papers, matched results will be shown. In addition to information extracting by keywords, the funRiceGenes database allows searching by gene symbol and genomic locus from either MSU or RAPdb (e.g., LOC_Os07g15770 or Os05g0158500), as the funRiceGenes database builds the associations between genomic locus of a gene and related published papers. Besides, funRiceGenes also lists all the genes related to a specified publication, which provides another option for information retrieving. We discussed this in the first paragraph of the Discussion section in the revised manuscript.

Reviewer: 2
The authors have created a new database, funRiceGenes, which contains functional information of rice genes and some other related data. The data were first collected from other databases and manually curated. The database is possibly useful, but I have some serious concerns as follows:
Oryzabase, which was created in 2000 and is still actively maintained, harbors a large amount of literature information. https://shigen.nig.ac.jp/rice/oryzabase/about/oryzabase
Though the data of Oryzabase are all curated, the authors seemed to re-curate them, and I don't understand why this was needed and what really had to be done.
Response: A number of genes archived in Oryzabase are merely members of gene families identified by bioinformatics analysis. We need to separate them from genes functionally characterized by experiments. In addition, Oryzabase also contains quantitative trait loci (QTL) associated with agronomic traits and assigns gene symbols to these QTL (https://shigen.nig.ac.jp/rice/oryzabase/gene/advanced/list). However, the casual gene of these QTL has not been identified yet. Thus these “genes” should be distinguished from genes functional characterized by experiments. In addition, we re-curated all the data collected from the China Rice Data Center and the Oryzabase database as a double-check to make sure all the information in our database is correct. And we did find some error information in the two databases.

While the database of the Michigan State Univ is virtually abandoned without new updates since 2013, Oryzabase and RAP-DB have been releasing newly curated hundreds or thousands of data every year. The authors' data that were "collected until 13 Feb 2014" (page 5) are very old and my feeling is that the researchers should need much fresher information.
Response: We updated the funRiceGenes database every two weeks since its initial construction in 2014. Since 2014, this database was updated using a Shiny application by tracking publications from PubMed and new records in the China Rice Data Center and Oryzabase databases. All updated records are available at https://funricegenes.github.io/news/, with the latest update performed on Sep 20th, 2017. We will keep updating of the funRiceGenes database in future.

First of all, the authors should mention that there are other efforts of extensive data curation of the rice genes. And, the authors should clearly state what are new and different from Oryzabase and RAP-DB in their database. Some clear example where functional descriptions were improved by the authors' effort should be shown.
Response: Many thanks for your valuable suggestions. We discussed the features of funRiceGenes and difference of this database from Oryzabase and RAP-DB in the first

paragraph of the Discussion section of the revised manuscript. We also clearly indicated the efforts of data curation from other database in the Background (page 3 line 11-16) and Result section (page 4 line 23-25).

Compared with Oryzabase and RAPdb, funRiceGenes has the following improvements:

1. The symbols of genes collected in funRiceGenes are much more accurate.
2. We separated member of gene families from functional characterized rice genes in the funRiceGenes database. A number of genes archived in Oryzabase and RAPdb are merely member of reported rice gene families identified by bioinformatics analysis rather than genes functionally characterized by experiments.
3. Some of the “genes” archived in Oryzabase are uncloned QTL rather than functionally characterized genes. The casual gene for the QTL has not been identified. We filtered these “genes” when we built the funRiceGenes database.
4. User-friendly query interface and tidy data for downloading are provided in the funRiceGenes database.

funRiceGenes also provides several additional functions:

1. Brief descriptions of the functions of collected genes and the supporting evidences are provided in the funRiceGenes database.
2. The interactions between different genes and the supporting evidences are provided in the funRiceGenes database.
3. Live update of the database every two weeks.

As I noted, the data of MSU are somewhat obsolete, but the authors' analyses depended heavily upon such data. Isn't it necessary to use up-to-date information?

Response: We totally agree with you that it is necessary to use up-to-date information. Thus we use not only the data from MSU, but also up-to-date data from other database.

We used the information of orthologous groups of seven plants from MSU, which is absent from the database of RAPdb and Oryzabase. We also used the data of gene families from both MSU and Oryzabase to build our database, to make a more comprehensive collection.

In addition, both MSU and RAPdb provide annotation of the Nipponbare reference genome, which are extensively used by a wide range of researchers. For each gene in the funRiceGenes database, both the MSU and the RAPdb genomic locus are provided for convenience of researchers.
