# Supplementary material for: funRiceGenes dataset for comprehensive understanding and application of rice functional genes
Source: Gigascience. 2017 Dec 4;7(1):gix119. doi: 10.1093/gigascience/gix119 (PMC5765555; doi:10.1093/gigascience/gix119)
Supplement: Response_to_Reviewer_Comments_Revision_1.pdf [file gix119_response_to_reviewer_comments_revision_1.pdf]

Author's Response To Reviewer Comments

Dear editors,
We have revised the format of our manuscript to adhere to the guidelines for a "Technical Note". We also registered our database resource in the SciCrunch.org database and included the RRID number in the result.

Sincerely,
Wen Yao, Ph. D.
National Key Laboratory of Crop Genetic Improvement
Huazhong Agricultural University
Wuhan 430070, China

Yidan Ouyang, Ph. D.
Professor, College of Life Science and Technology
National Key Laboratory of Crop Genetic Improvement
Huazhong Agricultural University
Wuhan 430070, China
