# Supplementary material for: funRiceGenes dataset for comprehensive understanding and application of rice functional genes
Source: Gigascience. 2017 Dec 4;7(1):gix119. doi: 10.1093/gigascience/gix119 (PMC5765555; doi:10.1093/gigascience/gix119)
Supplement: Reviewer_1_Report_(Original_Submission) -- Andy Pereira — 13 Aug 2017 Reviewed [file gix119_reviewer_1_report_(original_submission).pdf]

Reviewer Report

Title: funRiceGenes dataset for comprehensive understanding and application of rice functional genes

Version: Original Submission **Date:** 8/13/2017

Reviewer name: Andy Pereira

Reviewer Comments to Author:

The manuscript provides an integration of publicly available information on rice gene functions and associated attributes from heterogenous sources, in order to make the information available for biological interpretation.
A number of search tools have been developed or applied to derive associations between heterogeneous data subjects. These associations have also been used to derive networks of functional associations from literature that can provide a basis for further searches.
The interactive search page with a Shiny application for updating was tested with a number of genes of interest, and they made links between loci numbers and new publications, providing a potential gene function from available literature. I see that as a very good tool to test data and hypotheses in a research. Although the interactive page is a bit slow, and might be even more with more traffic from searches, it is user friendly and would be an asset for researchers doing GWAS or gene function identification. The utility for gene function information goes beyond Gramene and RAPdb, but will only be able to remain so if the planned automatic updates to the database remain functional.
Since the Nipponbare genome basis and annotation is used, is there a potential to survey overlapping genomic intervals from the indica genome sequences and make predictions of intervening syntenic genes?
Is the search scalable to use larger datasets or gene lists rather than individual genes to derive hypotheses from experimental data, eg what would be the pathways affected from mutation of a specific candidate gene, when no experimental data is available? Or, could one predict candidate genes that might perturb/affect a specific biological process. The availability of other network-based predictive methods and integration into funRiceGenes would be able to provide further tools for experimenters.
The funRiceGenes application on publications has similarities to the Textpresso application for many model systems from Arabidopsis (http://www.textpresso.org/arabidopsis/) to mouse and also initiated for Oryza sativa (http://map.lab.nig.ac.jp:8095/textpresso/index.html). This rice functional genomics application funRiceGenes should be shown how it distinguishes from the textpresso tool with differences outlined in the manuscript.

Methods

Are the methods appropriate to the aims of the study, are they well described, and are necessary controls included? Yes

Conclusions

Are the conclusions adequately supported by the data shown? Yes

Reporting Standards

Does the manuscript adhere to the journal's guidelines on minimum standards of reporting? YesChoose an item.

Statistics

Are you able to assess all statistics in the manuscript, including the appropriateness of statistical tests used? There are no statistics in the manuscript.

Quality of Written English

Please indicate the quality of language in the manuscript: Acceptable

Declaration of Competing Interests

Please complete a declaration of competing interests, considering the following questions:

- Have you in the past five years received reimbursements, fees, funding, or salary from an organisation that may in any way gain or lose financially from the publication of this manuscript, either now or in the future?
- Do you hold any stocks or shares in an organisation that may in any way gain or lose financially from the publication of this manuscript, either now or in the future?
- Do you hold or are you currently applying for any patents relating to the content of the manuscript?
- Have you received reimbursements, fees, funding, or salary from an organization that holds or has applied for patents relating to the content of the manuscript?
- Do you have any other financial competing interests?
- Do you have any non-financial competing interests in relation to this paper?

If you can answer no to all of the above, write 'I declare that I have no competing interests' below. If your reply is yes to any, please give details below.

I declare that I have no competing interests

I agree to the open peer review policy of the journal. I understand that my name will be included on my report to the authors and, if the manuscript is accepted for publication, my named report including any attachments I upload will be posted on the website along with the authors' responses. I agree for my report to be made available under an Open Access Creative Commons CC-BY license (http://creativecommons.org/licenses/by/4.0/). I understand that any comments which I do not wish to be included in my named report can be included as confidential comments to the editors, which will not be published.

I agree to the open peer review policy of the journal

To further support our reviewers, we have joined with Publons, where you can gain additional credit to further highlight your hard work (see: https://publons.com/journal/530/gigascience). On publication of this paper, your review will be automatically added to Publons, you can then choose whether or not to claim your Publons credit. I understand this statement.

Yes
