# Supplementary material for: funRiceGenes dataset for comprehensive understanding and application of rice functional genes
Source: Gigascience. 2017 Dec 4;7(1):gix119. doi: 10.1093/gigascience/gix119 (PMC5765555; doi:10.1093/gigascience/gix119)
Supplement: Reviewer_2_Report_(Original_Submission) -- Takeshi Itoh — 16 Aug 2017 Reviewed [file gix119_reviewer_2_report_(original_submission).pdf]

Reviewer Report

Title: funRiceGenes dataset for comprehensive understanding and application of rice functional genes

Version: Original Submission **Date:** 8/16/2017

Reviewer name: Takeshi Itoh

Reviewer Comments to Author:

The authors have created a new database, funRiceGenes, which contains functional information of rice genes and some other related data. The data were first collected from other databases and manually curated. The database is possibly useful, but I have some serious concerns as follows:

Oryzabase, which was created in 2000 and is still actively maintained, harbors a large amount of literature information.
https://shigen.nig.ac.jp/rice/oryzabase/about/oryzabase
Though the data of Oryzabase are all curated, the authors seemed to re-curate them, and I don't understand why this was needed and what really had to be done.

While the database of the Michigan State Univ is virtually abandoned without new updates since 2013, Oryzabase and RAP-DB have been releasing newly curated hundreds or thousands of data every year. The authors' data that were "collected until 13 Feb 2014" (page 5) are very old and my feeling is that the researchers should need much fresher information. First of all, the authors should mention that there are other efforts of extensive data curation of the rice genes. And, the authors should clearly state what are new and different from Oryzabase and RAP-DB in their database. Some clear example where functional descriptions were improved by the authors' effort should be shown.

As I noted, the data of MSU are somewhat obsolete, but the authors' analyses depended heavily upon such data. Isn't it necessary to use up-to-date information?

Methods

Are the methods appropriate to the aims of the study, are they well described, and are necessary controls included? Yes

Conclusions

Are the conclusions adequately supported by the data shown? Yes

Reporting Standards

Does the manuscript adhere to the journal’s guidelines on minimum standards of reporting? YesChoose an item.

Statistics

Are you able to assess all statistics in the manuscript, including the appropriateness of statistical tests used? There are no statistics in the manuscript.

Quality of Written English

Please indicate the quality of language in the manuscript: Needs some language corrections before being published

Declaration of Competing Interests

Please complete a declaration of competing interests, considering the following questions:

- Have you in the past five years received reimbursements, fees, funding, or salary from an organisation that may in any way gain or lose financially from the publication of this manuscript, either now or in the future?
- Do you hold any stocks or shares in an organisation that may in any way gain or lose financially from the publication of this manuscript, either now or in the future?
- Do you hold or are you currently applying for any patents relating to the content of the manuscript?
- Have you received reimbursements, fees, funding, or salary from an organization that holds or has applied for patents relating to the content of the manuscript?
- Do you have any other financial competing interests?
- Do you have any non-financial competing interests in relation to this paper?

If you can answer no to all of the above, write 'I declare that I have no competing interests' below. If your reply is yes to any, please give details below.

I declare that I have no competing interests

I agree to the open peer review policy of the journal. I understand that my name will be included on my report to the authors and, if the manuscript is accepted for publication, my named report including any attachments I upload will be posted on the website along with the authors' responses. I agree for my report to be made available under an Open Access Creative Commons CC-BY license (http://creativecommons.org/licenses/by/4.0/). I understand that any comments which I do not wish to be included in my named report can be included as confidential comments to the editors, which will not be published.

I agree to the open peer review policy of the journal

To further support our reviewers, we have joined with Publons, where you can gain additional credit to further highlight your hard work (see: https://publons.com/journal/530/gigascience). On publication of this paper, your review will be automatically added to Publons, you can then choose whether or not to claim your Publons credit. I understand this statement.

Yes
